# A Network Pharmacology Analysis to Explore the Effect of* Astragali Radix-Radix Angelica Sinensis* on Traumatic Brain Injury

**DOI:** 10.1155/2018/3951783

**Published:** 2018-11-25

**Authors:** Genggeng Xie, Weijun Peng, Pengfei Li, Zian Xia, Yuanyuan Zhong, Feng He, Yimingaji Tulake, Dandan Feng, Yang Wang, Zhihua Xing

**Affiliations:** ^1^Institute of Integrative Medicine, Xiangya Hospital, Central South University, Changsha 410008, Hunan, China; ^2^Institute of Integrative Medicine, 2nd Xiangya Hospital, Central South University, Changsha 410008, Hunan, China; ^3^Department of Hepatobiliary Surgery, Xiangya Hospital, Central South University, Changsha 410008, Hunan, China; ^4^Department of General Surgery, Xinjiang Production and Construction Corps Third Division Hospital, Xinjiang 844000, China

## Abstract

Traumatic brain injury (TBI) is a critical public health and socioeconomic problem worldwide. The herb pair* Astragali Radix* (AR)-*Radix Angelica Sinensis* (RAS) is a common prescribed herbal formula or is added to other Chinese medicine prescriptions for traumatic brain injury (TBI) treatment. However, the underlying mechanisms are unclear. In this study, we aimed to explore the active ingredients and action targets of AR-RAS based on the combined methods of network pharmacology prediction and experimental verification. Furthermore, the corresponding potential mechanisms of “multicomponents, multitargets, and multipathways” were disclosed.* Methods*. A network pharmacology approach including ADME (absorption, distribution, metabolism, and excretion) filter analysis, target prediction, known therapeutic targets collection, Gene Ontology (GO), pathway enrichment analysis, and network construction was used in this study. Further verification experiments were performed to reveal the therapeutic effects of AR-RAS in a rat model of TBI.* Results*. The comprehensive systematic approach was to successfully identify 14 bioactive ingredients in AR-RAS, while 33 potential targets hit by these ingredients related to TBI. Based on GO annotation analysis, multiple biological processes were significantly regulated by AR-RAS. In addition, 89 novel signaling pathways (P<0.05) underlying the effects of AR-RAS for TBI treatment were identified by DAVID. The neurotrophin signaling pathway was suggested as the major related pathway targeted by AR-RAS to improve axonal growth. The animal experiment confirmed that AR-RAS significantly induced tissue recovery and improved neurological deficits on the 14th day (P<0.01). Treatment with AR-RAS markedly reduced the protein and mRNA expression level of NogoA in the hippocampus of TBI rats.* Conclusion*. Our work illuminates the “multicompounds, multitargets, and multipathways” curative action of AR-RAS in the treatment of TBI by network pharmacology. The animal experiment verifies the effects of AR-RAS on neurological function improvement and axonal outgrowth via downregulation of NogoA expression, providing a theoretical basis for further research on treatment of TBI.

## 1. Introduction

Traumatic brain injury (TBI) is described as any traumatically induced structural injury or physiological disruption of brain function as a result of an external force. It is an important contributing factor of morbidity and mortality in adult and pediatric population worldwide [1, 2]. Recently, substantial evidence confirms that the annual global incidence and prevalence rate of TBI increased progressively over the past years [3]. It seriously damages people's quality of life and results in a massive economic toll [4–6]. TBI is a highly complex disorder that includes varying degrees of contusion, diffuse axonal injury, hemorrhage, and hypoxia [7, 8]. Neuropathological studies have shown that TBI affects structural brain networks progressively, with focal axon alteration to delay axonal disconnection [9, 10]. Despite various efforts, currently, there is no therapeutic option available to ease or prevent nerve dysfunction after TBI [11, 12]. On account of the fact that TBI involves complicated pathological mechanisms of multiple cellular and molecular events [13, 14], including blood-brain barrier breakdown, neuronal death, and neurodegeneration, thus, “one-compound, one-target, and one-pathway” based modern drugs have poor curative effect.

Traditional Chinese medicine (TCM) has the characteristics of “multicomponents, multitargets, and multipathways” and generates attractive effect on TBI treatment and rehabilitation [15–18]. Astragalus herbal extract has been declared to reverse memory loss and prevent the loss of axons and synapses in the cerebral cortex and hippocampus in mice [19]. Radix Astragali provides significant protection against brain injury in various models of oxidative stress-related disease, possessing antioxidant activity [20]. Radix Astragali shows markedly neuron protection in immature brain cortex after hypoxia/ischemic brain damage [21, 22]. Radix angelica sinensis has been known for its active components in possessing wide pharmacological activities, such as memory amelioration and neuroprotective [23–25]. Based on the above, we considered AR-RAS could be used as an effective therapeutic for repairing and protecting nerve after TBI. In addition, molecular details about how AR-RAS affects TBI are still unclear.

Network pharmacology is rapidly becoming a cutting-edge research field in contemporary drug studies [15]. In recent years, there was an increasing concern about applying the network pharmacology to reveal the scientific basis and systematic features of TCM. Numerous active chemical compositions of TCM target multiple proteins in the biological network of some disease [26]. Network pharmacology allows us to form an initial understanding of how the multiple ingredients in an herbal formula act in synergy, and what effect they can have on multiple targets of a disease [27]. It is used not only to explore the pharmacological activity of a single compound drug, but also to examine combination therapy [28]. It is also a promising method for discovering potential drugs from herbal medicine [29]. The network builds a link between drugs, proteins, and pathways and interprets the relationship between them [30].

NogoA, a myelin-rich membrane protein, is a potent neurite outgrowth inhibitor and noted as an inhibitor blocking axonal regrowth and plasticity after CNS (Central Nervous System) injuries [31, 32]. In the present study, a network pharmacology approach was employed to identify the active ingredients of AR-RAS and potential targets when it is utilized to treat TBI. The effects of AR-RAS on axonal repair and regulation of axon growth inhibitory factors NogoA in TBI mice were explored. The results may provide a scientific basis for TCM to preserve and promote the regeneration of nerve axons in TBI.

## 2. Materials and Methods

### 2.1. Network Pharmacology-Based Prediction of the Potential Actions of AR-RAS on TBI

#### 2.1.1. Application of Databases

Databases include Chinese medicine system pharmacology technology platform (TCMSP, http://lsp.nwu.edu.cn/tcmspsearch.php); UniProt protein knowledgebase (http://www.uniprot.org); DAVID Functional Annotation Bioinformatics Microarray Analysis (https://david.ncifcrf.gov/summary.jsp); Therapeutic Target Database (TTD, http://bidd.nus.edu.sg/group/cjttd); Online Mendelian Inheritance in Man (OMIM, http://www.omim.org/), and Human Protein Reference Database (HPRD, http://www.hprd.org/).

#### 2.1.2. Molecular Database Building

The ingredients of both AR and RAS were obtained from TCMSP database, which provided information on various active ingredients related to TCM drugs and their target proteins. TCMSP provides detailed, up-to-date, and accurate structural and physicochemical properties like molecular weight, oral bioavailability, drug likeness, intestinal epithelial permeability and aqueous solubility, drug targets and their relationships with diseases [33]. In order to gather all available information about ingredients of herbal medicines, we performed an extensive literature search for each herbal medicine. Because of the nonstandard naming, we input the protein names with the species limited to “homo sapiens” and we could receive their official symbol through UniProtKB.

#### 2.1.3. Oral Bioavailability Screening and Drug-Likeness Evaluation

Due to the shortcomings of biological experiments including time-consumption and high-cost, the identification of ADME (absorption, distribution, metabolism, and excretion) properties using silico tools has now become an inevitable paradigm in pharmaceutical research. In this study, two ADME-related models, the evaluation of oral bioavailability (OB) and drug likeness (DL), are employed to identify the potential bioactive compounds of AR-RAS. Oral route, the principal drug delivery system, is commonly used in the administration of herbal medicines. OB, the percentage of an oral dose able to produce a pharmacological activity, is one of the most desirable attributes of a new drug [34]. In this work, OB was measured by a novel and robust in-house system OBioavail 1.1 that integrated the metabolism (P450 3A4) and transport (P-glycoprotein) information [35]. Those components with OB ≥ 30% were selected as the candidate molecules for further study [36]. Database-dependent DL evaluation approach based on Tanimoto coefficient was applied and shown as

(1) $$\begin{array}{c} T\left(\;A,B\;\right)=\frac{\left(A\times B\right)}{\left(\left|A\right|2+\left|B\right|2-A\times B\right)} \end{array}$$

In this equation, A represents the molecular descriptors of herbal compounds, and B displays the average molecular properties of all compounds in DrugBank [37]. In this work, compounds with DL ≥ 0.18 were selected as the candidate bioactive molecules [38]. These ingredients, which met all of two criteria above, were selected as candidate molecules for additional analysis.

#### 2.1.4. TBI-Specific Protein Collecting

The known therapeutic target proteins of TBI were screened from the Therapeutic Target Database (TTD) and Online Mendelian Inheritance in Man database (OMIM). TTD is a publicly accessible database which provides comprehensive information about the known therapeutic protein and nucleic acid targets described in the literature, the targeted disease conditions, the pathway information and the corresponding drugs/ligands directed at each of these targets [39]. OMIM catalogs all known diseases with a genetic component and links them to the relevant genes in the human genome, which provides references for further research and tools for genomic analysis of cataloged genes [40].

#### 2.1.5. Protein-Protein Interaction Data

The proteins acquired from TTD and OMIM were used as hub proteins and submitted to Human Protein Reference Database (HPRD) to acquire the proteins interacting with these hub proteins. HPRD is a resource for experimentally derived information about the human proteome including protein-protein interactions, posttranslational modifications (PTMs), and tissue expression [41].

#### 2.1.6. Enrichment Analysis

Gene ontology (GO) and Kyoto Encyclopedia of Genes and Genomes (KEGG) pathway enrichment analyses were also performed on the target data, using the Database for Annotation, Visualization and Integrated Discovery (DAVID). P values were derived from the DAVID database and are modified Fisher exact P values. Smaller P values indicated greater enrichment. Only functional annotations having the enrichment P values corrected by both algorithms Bonferroni and Benjamini (P < 0.05) were chosen for further analysis.

#### 2.1.7. Network Construction

Network construction was performed as follows: (1) active compound-target network of AR-RAS; (2) active compound-pathway of AR-RAS; (3) the PPI data obtained to construct TBI-specific protein interaction network; (4) potential compounds, putative targets from AR-RAS for treating TBI were used to build a potential compound-potential target-pathway (pC-pT-P) network.

Cytoscape3.4.6, which is widely used in network pharmacology research, is used to visualize biological pathways and intermolecular interaction networks, among others [42]. Furthermore, it supplies a basic set of features for data integration, analysis, and visualization for complicated network analysis.

### 2.2. Animal Experiment

#### 2.2.1. Animals

SPF-grade SD male rats weighing 220-250g (6-8 weeks) were recruited from Hunan Silaike Jingda Experimental Animal Co., Ltd. (Changsha, China). They were housed in Department of Laboratory Animals, Central South University (Changsha, China). The environment was controlled suitably (12-hour light/dark cycle, room temperature at 25°C and 50 ± 10% relative humidity). The rats were subdivided into sham, TBI model, and AR-RAS treated groups randomly (n=5 rats in each group).

#### 2.2.2. Astragali Radix (AR)-Radix Angelica Sinensis (RAS) Decoction Preparation

Astragali Radix (AR) (Specimen No. 2016041687) and Radix Angelica Sinensis (RAS) (No. 201604188) were purchased from TCM pharmacy of Xiangya Hospital. The herbals were later certified by experts and in accordance with the Chinese Pharmacopoeia. The exact amounts of Astragali Radix (AR) and Radix Angelica Sinensis (RAS) were weighed depending on a ratio of 5:1 and then mixed well. Chinese medicine dosage for the rats (200 g) was calculated in proportion to humans (70 kg) using body surface area conversion; each gavage was 3.24 g/kg·d. The herbs were immersed in 8 volumes of deionized water for 0.5 h and boiled for 1 h. The sample was filtered, and 6 volumes of deionized water were added to the residue for the second extraction. The two filtrates were merged containing 1 g of crude herb per milliliter in final. It was stored at 4°C before use.

#### 2.2.3. Controlled Cortical Impact Model of TBI and Administration of Drugs

The rats were intraperitoneally anesthetized with 3% pentobarbital (50 g/kg). All operations were conducted in a sterile environment. A midline incision was done to expose the skull between bregma and lambda suture lines. A 5 mm craniotomy was performed, with dental drill, lateral to the central fissure on the left side of the skull centered between lambda and bregma. Sham animals were only the same craniotomy but did not receive an impact on the brain. The injury was made by an automated controlled pneumatic impact device (PSI TBI-0310 Impactor, Precision Systems & Instrumentation, Fairfax Station, VA). The parameters consisted of a 5 mm deep contusion, impact velocity 6.0 m/sec, and dwell time 500 msec [43, 44]. After the injury, the incision was closed with interrupted 3-0 silk sutures, and the animal was placed into an electric blanket to maintain normal core temperature for 45 min after injury. Then animals were placed in a clean cage. CCI rats of AR-RAS treated group received AR-RAS decoction (3.24 g/kg) orally once a day; sham and TBI groups were administered 0.9% normal saline by volume equal to AR-RAS treated group. The gavage was continuous for 14 days.

#### 2.2.4. Modified Neurological Severity Score (mNSS) Test

Posttraumatic neurological impairments were measured using mNSS test at 1st, 3rd, 7^th^, and 14th after TBI. The mNSS was a behavioral test including the motor tests (6 points), sensory tests (2 points), beam balance tests (BBT) (6 points), reflexes absent, and abnormal movements (4 points). In the test, each score point was awarded for the inability to perform the test or for the lack of a tested reflex. Neurological function was graded on a scale of 0–18, where a total score of 18 points indicates severe neurological deficit and a score of 0 indicates normal performance.

#### 2.2.5. Sample Preparation

On 14th day after TBI, the animals were intraperitoneal anesthetic injected, perfused with 200 ml of cold 0.9% normal saline from the heart into the aorta, and decapitated in cervical vertebrae. The hippocampus of the injured side was dissected immediately and placed into cryopreservation tubes, stored in liquid nitrogen, and stored at −80°C until analysis of mRNA with RT-qPCR and protein with western blotting. The rest of brain tissue was removed and post-fixed in 4% paraformaldehyde overnight. Specimens next underwent gradient alcohol dehydration and were paraffin-embedded.

#### 2.2.6. Hematoxylin-Eosin Staining

Five micrometers of the coronal sections were cut. The paraffin sections were subjected to dewaxing and hydration and then stained with hematoxylin-eosin (HE). Briefly, coronal sections were deparaffinized in Histo-clear and rehydrated through 100% to 70% graded ethanol to distilled water. Then the section stained with toluidine blue for 30 minutes and then 2 or 3 drops of glacial acetic acid. Pathological changes were noted under a light microscope.

#### 2.2.7. Western Blot Analysis

The hippocampal tissues were mechanically lysed in 300 ul Radio Immunoprecipitation Assay Lysis Buffer for 30 min on ice. The lysates were centrifuged at 12,000 g for 15 minutes at 4°C, and supernatants were collected. The protein concentration was estimated by BCA assay. 50 ug of proteins were loaded and separated on 10% separating gel and 5% stacking gel for SDS-polyacrylamide gel electrophoresis (PAGE) and subsequently transferred to the polypropylene fluoride (PVDF) membrane. Blots were blocked for 1 h in a Tris-buffered saline solution with 0.1% Tween-20 (TBST) and 5% skim milk and then incubated overnight at 4°C with a primary rabbit anti-NogoA (1:5,000; Abcam, Cambridge, UK; catalog no. ab62024). Blots were probed simultaneously with mouse anti-*β*-actin (1:5,000; Proteintech, USA; catalog no. 60008-1-Ig) as a loading control. Following wash, the membranes were incubated with horse radish peroxidase (HRP)-labeled anti-rabbit IgG (1:6,000) as the secondary antibody for 1h at room temperature. Then the membranes were visualized with an enhanced chemiluminescence kit. The ratios of target proteins for internal control were calculated using ImageJ software version 1.5.0.

#### 2.2.8. Quantitative RT-qPCR

The total RNA was extracted from hippocampus tissues using TRIzol reagent (Invitrogen) following the manufacturer's instructions. RNA concentration was monitored with the ultraviolet spectrophotometer. The ratio of OD 260 to OD 280 was set between 1.8 and 2.0. 2*μ*g of RNA was reversely transcribed into cDNA with Reverse Transcription assay kit, according to manufacturer's instructions. With NCBI Genebank and Primer 5.0 software, primer sequences for NogoA and *β*-actin were designed and synthesized by Shanghai Sangon Biotechnology Co. Ltd. The gene sequences of primers are presented in Table 1.

Amplification of RNA was performed using SYBR PCR kit (Sigma Invitrogen, USA). A Real-Time PCR machine is set for 40 cycles amplifying the RNA: 95°C for 10min, 95°C for 15s, and 60°C for 50s. A melt curve is from 65°C to 95°C. *β*-actin mRNA served as an internal control. The relative normalized expression of NogoA was recorded and analyzed using the 2^−ΔΔCt^ method.

#### 2.2.9. Statistical Analysis

Statistical analysis was performed using GraphPad Prism 5.0 software. All data are expressed as Mean ± SD. Data from mNSS was analyzed by two-way analysis of variance (ANOVA). Statistical differences of the remaining biochemical data were determined using one-way ANOVA. A value of p<0.05 was considered statistically significant.

## 3. Results

### 3.1. Treatment with AR-RAS Decreased Neurological Function Scores of TBI Rats

**Figure 1:**
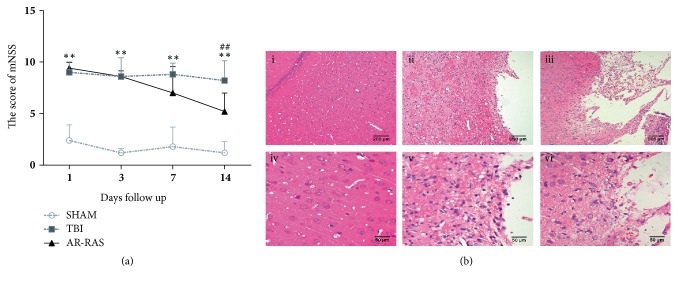
In vivo effects of AR-RAS on TBI. (a) Temporal profile of mNSS in saline and AR-RAS treated rats after TBI. It is shown that mNSS score is reduced significantly in the AR-RAS treated group on Day 14 (P < 0.01) after TBI compared to the TBI group. Data are presented as Mean ± SD (n = 5). *∗∗*P < 0.001 (TBI vs. sham), ^##^P< 0.001 (TBI vs. AR-RAS). (b) The morphology and structure of nerve cells in brain tissue in different groups. The top row pictures are HE × 100 (i, ii, iii), the bottom row pictures are HE × 400 (iv, v, vi). (i) and (iv) are sham group, (ii) and (v) are TBI group, and (iii) and (vi) are AR-RAS group.

Neurological functional deficits caused by injury in the left hemispheric cortex of rats were monitored by mNSS (Figure 1(a)). The TBI group showed significant functional deficits in the 1st, 3rd, 7^th^, and 14th day after TBI, compared with the sham group (P<0.01). The difference of neurological function score between in the TBI group and AR-RAS group was not statistically significant until the 14th day. AR-RAS treatment significantly cut the mNSS compared to the TBI group at day 14 (P<0.01) after TBI.

### 3.2. Treatment with AR-RAS of TBI Rats Significantly Attenuated Tissue Damage

As shown in Figure 1(b), under the microscope, the brain tissues of the sham group had clear and dense structures, and their neuronal structures were normal. Neurons of brain tissue in sham group were arranged uniformly, accompanied by the abundant cytoplasm and numerous normal neurocytes. In contrast, TBI group showed loosely structured brain tissue; swollen neural cells had extensive vacuolar changes. The cytoplasmic content was decreased; cellular necrosis and vacuolization were present. After treatments of AR-RAS, the edema and necrosis in lesioned zones of the brain were significantly attenuated, and neural cell number was effectively increased when compared with the TBI group. In addition, a few normal neurons were visible in AR-RAS-treated group.

### 3.3. Active Components and Target Identification of AR-RAS

Totally, 87 components of AR and 125 components of RAS were collected from the TCMSP database and literature (as showed in Table S1). A total of 19 compounds (as displayed in Table 2) of AR-RAS were selected when OB ≥ 30% and DL≥ 0.18. And 226 potential targets from the 19 compounds were generated from the TCMSP database. The detailed data of the targets is given in Table S2.

### 3.4. C-T Network Construction and Enrichment Analysis of AR-RAS

**Figure 2:**
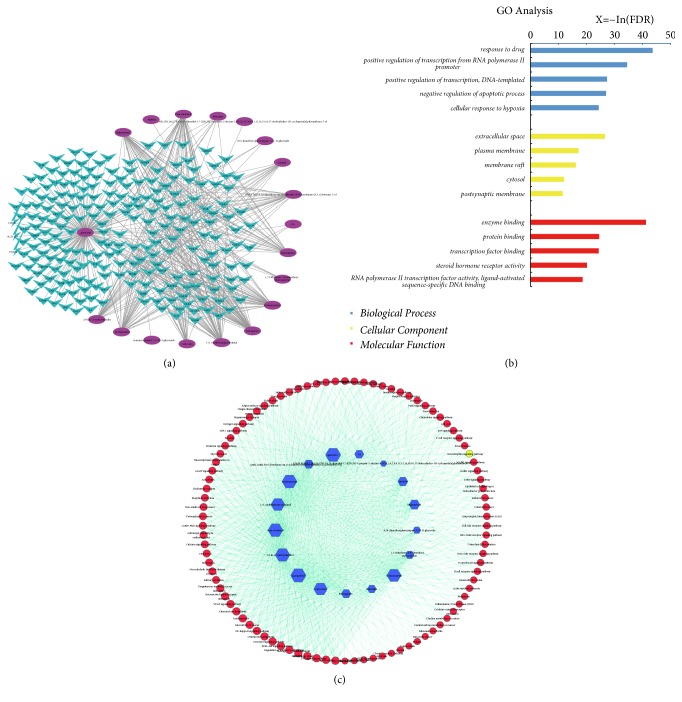
A network analysis of AR-RAS. (a) The compound-putative protein network is constructed by linking the 19 candidate compounds and their putative 226 target proteins. The nodes represent candidate compounds in AR-RAS which are shown as purple oval, and the targets are indicated by green fusiform (245 nodes and 517 edges). (b) Gene Ontology (GO) analysis of targets. Top 5 significantly enrichment terms in Biological Process (BP), Cellular Component (CC), and Molecular Function (MF). Longitudinal-axis shows target proteins significantly enrich in Biological Process, Cellular Component, and Molecular Function categories of the targets, and the horizontal-axis shows the enrichment score of these terms (-Log10(P)). The blue bar represents Biological Process, red bar represents Molecular Function, and yellow bar represents Cellular Component. (c) Compound-pathway network. Blue hexagon: a compound of AR-RAS and red and yellow circles: target pathway. Edge: interaction between a target and a pathway.

19 compounds and 226 target proteins were imported to Cytoscape 3.4.6 software. The “active component-target interaction” of AR-RAS was plotted interaction network diagram, which consists of 245 nodes and 527 edges (Figure 2(a)). It reflected the complex network relationship between “one ingredient-multiple targets” and “one target-multiple ingredients.” It was found that the target genes of AR-RAS on active component prediction were mainly significantly enrichened in 624 Biological Processes (BP), 127 Molecular Functions (MF), and 70 Cell Components (CC). As shown in Figure 2(b), in biological processes, the top 5 functions of target proteins were response to drug, positive regulation of transcription from RNA polymerase II promoter and positive regulation of transcription, DNA-templated, antiapoptotic, response to hypoxia. In aspect of molecular functions, they were mainly enrichment in enzyme binding, protein binding, and transcription factor binding, etc. The target proteins existed in extracellular space, plasma membrane, membrane raft, cytosol and postsynaptic membrane, etc.

Signaling pathways, as an important component of the system pharmacology, link receptor-ligand interactions to pharmacodynamics outputs. The pathways significantly affected by AR-RAS were identified using public DAVID database, in which a compound and a signal pathway were linked if the compound targets on the proteins appeared in the signal pathways. These compounds were enriched in 95 signal pathways (P < 0.001) (Table S3). The compound-pathway network was presented in Figure 2(c). It is verified that AR-RAS exerts its function in the way of multipathway, multitarget and overall cooperation.

### 3.5. Analyses on TBI Based Specific Protein Interaction Network

**Figure 3:**
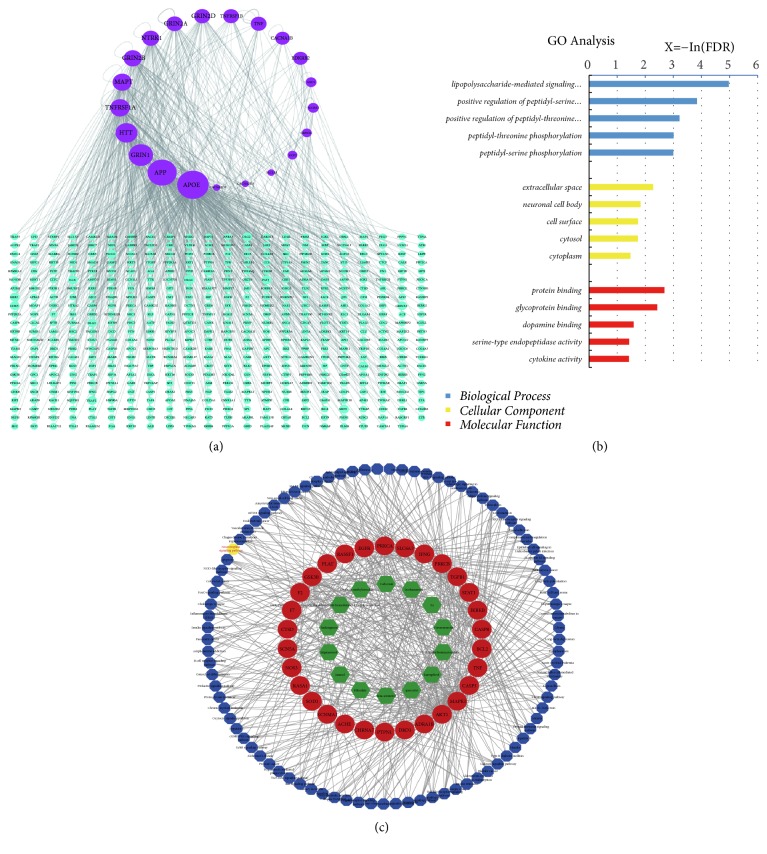
TBI-related protein interaction network. (a) Protein-protein interaction network. 21 hub proteins (purple circular) are determined through the analysis of Therapeutic Target Database (TTD) and Online Mendelian Inheritance in Man database (OMIM). 505 proteins (blue circular) are acquired from HPRD. The size of a node is proportional to the value of a degree. (b) Gene Ontology (GO) analysis of TBI-related proteins of AR-RAS. Longitudinal-axis shows significantly enriched Biological Process, Cellular Component, and Molecular Function categories of the targets, and the horizontal-axis displays the P value of these terms (-Log10(P)). The blue bar represents Biological Process, red bar represents Molecular Function, and yellow bar represents Cellular Component. (c) pC-pT-P network of AR-RAS for treating TBI. 30 candidate protein targets (red circle) of AR-RAS are screened for treating TBI. 14 active compounds (green hexagon) of AR-RAS are obtained for treating TBI. 89 target pathways (blue and yellow octagons) are enriched by the 30 proteins for treating TBI.

We screened the TBI-specific genes and protein targets OMIM and TTD databases. 21 TBI-specific genes/proteins were generated then submitted to Human Protein Reference Database (HPRD). The TBI-related protein-protein interaction (PPI) network is constructed in Figure 3(a). The network contains 526 nodes and 722 edges. The size of nodes is proportional to the value of the degree. The detail information of the TBI-specific proteins is shown in Table S4. The top 10 proteins of TBI-specific proteins according to the degree are set out in Table 3. Higher degree centrality indicates greater importance to TBI. This suggests that these genes may be the key or crucial genes involved in TBI progression.

### 3.6. Enrichment Analysis and pC-pT-P Network Construction of AR-RAS for Treating TBI

33 TBI potential target human proteins were targeted by 14 potential compounds from AR-RAS (Figure 3(c)), in which each component hit more than one target. It indicated the potential synergistic effect of the ingredients of AR-RAS on the protein targets. As showed in Figure 3(b), top five functions were lipopolysaccharide-mediated signaling pathway, positive regulation of peptidyl-serine phosphorylation, positive regulation of peptidyl-threonine, phosphorylation, peptidyl-threonine phosphorylation, and peptidyl-serine phosphorylation. After target validation, we tried to elucidate the mechanism of action of AR-RAS for treating TBI and identify the signal pathways related to protein targets of AR-RAS. In total, 89 signal pathways related to AR-RAS for treating TBI were identified, and they are presented in Table S5.

### 3.7. AR-RAS Upregulated the Protein and mRNA Expression Level of NogoA after TBI

**Figure 4:**
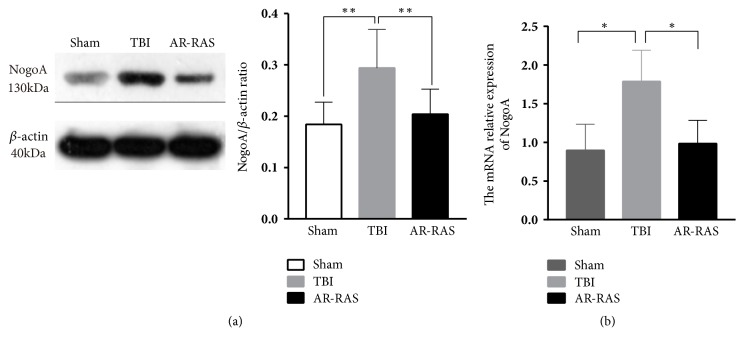
AR-RAS influence the protein expression and mRNA level of NogoA. (a) Graph shows the quantitative densitometry analyses of NogoA protein, with data expressed as Mean ± SD; *∗*p<0.05 and *∗∗*p<0.01 significantly different from TBI animals (N=5). (b) A Quantitative real-time PCR of mRNA expression of NogoA in sham and TBI and AR-RAS-treated groups on day 14 after TBI (N = 5). Bars show Mean ± SD. *∗*p<0.05, compared with TBI group.

Western blotting and RT-qPCR were used to quantify the protein and mRNA expression level of NogoA in the hippocampus tissues, with *β*-actin as an internal control (Figure 4). The result showed that NogoA protein significantly increased in the hippocampus of the TBI group compared to sham group on day 14 after injury (P<0.01). However, NogoA protein expression decreased in the hippocampus AR-RAS group than in the TBI group 14 days (Figure 4(a)). In the same way, the mRNA level of NogoA in the hippocampus of the TBI group was significantly upregulated in TBI group (P<0.05). And the mRNA level of NogoA was down regulated in AR-RAS-treated group on the 14th day after TBI, as compared to TBI rats (P<0.05) (Figure 4(b)).

## 4. Discussion

TCM network pharmacology provides a fresh perspective on the identification of active herbal compounds against specific diseases or pathological processes, as well as broader insights into the molecular mechanisms of the compounds. In the present study, a network pharmacology-based method was employed to elucidate the pharmacological mechanisms of AR-RAS to treat TBI. According to the above results, we assumed that bioactive compounds of AR-RAS might simultaneously interact with multiple pathways like HIF-1, MAPK, and neurotrophin signaling pathways, thereby exhibiting synergistic effects in TBI. The neurotrophin signaling pathway was suggested as the major related pathway targeted by AR-RAS to improve axonal growth. In fact, lots of researches have indicated the significant role of neurotrophin signaling pathways in promoting axonal growth.

We first confirmed that AR-RAS decoctum was effective in treating TBI* in vivo*. The results of mNSS and HE staining showed AR-RAS could improve nerve function defect and promote recovery of damaged neurons after TBI (Figure 1). This indicates that AR-RAS is available for treatment of TBI, being used alone or added to other TCM formulation for TBI. However, its “multicomponents” and “multitargets” features make it more difficult to decipher the molecular mechanisms of AR-RAS in the treatment of TBI from a systematic perspective if employing routine methods.

Then, we utilized a newly developed pharmacological approach to analyze the active compounds and therapeutic targets of AR-RAS. The relationship between targets and herbs reflects the multitarget characteristics, which are one of the most important directions in modern drug discovery [45]. The 19 compounds of AR-RAS were screened from TCMSP database and 222 target proteins were obtained. Except as shown in Figure 2(b), the targets of active ingredients mainly participate in these biological processes, such as antiapoptosis, inflammatory reaction, reaction to hypoxia, and signal transduction. They were enriched in extracellular space, synapse, and axon terminals where there were played molecular reactions such as enzyme binding, protein binding, and cytokine activation. In KEGG analysis, the signal pathways were mainly associated with diseases, signal transduction, and immune correlation. Tumor-related disease pathways were the most numerous in disease-related pathways, including prostate cancer, nonsmall cell lung cancer, pancreatic cancer, and bladder cancer. At present, previous studies have confirmed that AR-RAS has potential antitumor effects [46, 47]. Therefore, it can be speculated that AR-RAS will be useful for treatment of these tumor diseases, which are worthy of further study. Most results of forecast target, GO enrichment, and KEGG pathway analysis were in conformity with the known pharmacological effects reported in previous study [48]. It showed the accuracy of the predicted targets by network pharmacology. TNF, VEGF, toll-like receptor (TLR), MAPK, and NF-*κ*B signaling pathways were the most important of the main pathways capable of regulating anti-inflammatory, neuroprotective, and antioxidative effects.

The PPI related to TBI indicates these genes played their roles in TBI development (Figure 3(a)). Following up, we filtered 14 compounds from AR-RAS targeted 33 TBI-related proteins (Figure 3(c)). The most targeted compounds were displayed as follows: Quercetin (MOL000098, OB=46.43% and DL= 0.28) possesses anti-inflammatory effects in vivo by inhibiting oxidative stress and cytokine production [49, 50]. Calycosin (MOL000417, OB = 47.75% and DL = 0.24) displays therapeutic effects on diabetic complication that strikingly downregulated HUVEC TGF-beta1, ICAM-1, and RAGE expressions [51]. In addition, it has antitumor, neuroprotective, anti-inflammatory, and proangiogenesis effects [52]. Stigmasterol (MOL000439, OB = 43.83% and DL = 0.76) exhibits potent ameliorate ketamine-induced behavioral, biochemical and histopathological alterations in mice showing its potential effects in the management of psychotic symptoms. It revealed increased GABA and GSH levels and decreased dopamine, MDA, TNF-*α* levels, and ACHE activity [53]. These studies were consistent with the results obtained from the analysis of the active component target. Based on GO annotation analysis, 42 biological processes were meaningfully regulated by AR-RAS to treat TBI, including positive regulation of peptidyl-serine phosphorylation, positive regulation of peptidyl-threonine, phosphorylation, peptidyl-threonine phosphorylation, and peptidyl-serine phosphorylation. HIF-1*α* is thought to be one of the most crucial signaling molecules in tissue responses to hypoxia, as it regulates many genes that are important in promoting cell survival such as Erythropoietin [54], vascular endothelial growth factor [55]. It also plays an important role in neuronal survival and death in hypoxia [56]. MAPK signal pathway is a major signaling pathway involved in regulating the inflammation-related pathogenesis and neuroprotective effects [57]. Above all, this confirms the “multicompounds, multitargets” therapeutic actions of AR-RAS in the treatment of TBI.

**Figure 5:**
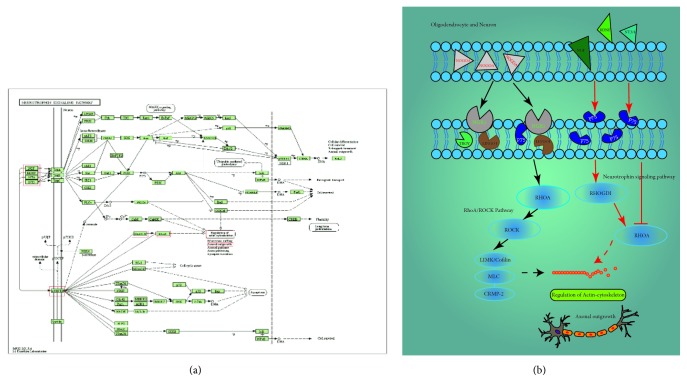
The mechanism of NogoA inhibits axonal growth. (a) Neurotrophin signal pathway. (b) Molecular mechanisms mediating NogoA-induced axonal growth inhibition and neurotrophin signaling pathway in axon growth.

Based on target identification and pathway analysis, we concentrated on neurotrophin signaling pathway, which was related to axon outgrowth (Figure 5(a)). NogoA is expressed mainly by oligodendrocytes on the axonal and outer myelin membrane. It mainly exerts neurite growth inhibitory function [58–61]. Molecular mechanisms mediating NogoA-induced axonal growth inhibition and neurotrophin signaling pathway in axon growth were shown in Figure 5(b). The study found that unliganded p75NTR promoted RhoA activation and neurotrophins such as BDNF, NGF, and NT 3/4 binding prevented this activation, thereby promoting neurite outgrowth [62]. Activation of RhoA by myelin proteins occurs through the recruitment of the Rho inhibitor RhoGDI to p75NTR, thereby releasing RhoA [63]. NogoA binds to its receptor NgR and the NgR1-associated proteins Lingo and p75 or Troy is stimulated by Nogo-66 leading to the intracellular activation of the RhoA/ROCK that prevents actin cytoskeleton polymerization in the growth cone and thereby blocks neurite extension [31]. The present animal experiment demonstrated that TBI rats showed a higher mRNA and protein expression of hippocampus NogoA, accompanied by relatively delayed deficiencies in neurological function. The elevated mRNA and protein level of NogoA in the hippocampus were reversed by the treatment of AR-RAS. AR-RAS treatment decreased brain tissue edema and protected and repaired nerve cells. In addition, neurological dysfunction of rats after TBI was enhanced by the treatment of AR-RAS. This was the first report on the axon regeneration of AR-RAS in an animal model of TBI. It may serve as a novel treatment option for cognitive impairment after TBI.

## 5. Conclusion

Our work illuminates the “multicompounds, multitargets, and multipathways” therapeutic action of AR-RAS in the treatment of TBI by network pharmacology, providing a potential novel method for treating TBI. Animal experiment verification expressed support for our findings. These results provided evidence that AR-RAS may promote recovery of neurological function and axonal outgrowth via neurotrophin signaling pathway and downregulate the expression of NogoA, which could be used as part of a novel therapeutic approach to treatment of TBI. Therefore, further pharmacologic studies and confirmation of prediction of AR-RAS are required.

## Figures and Tables

**Table 1:** Sequences of primers used for real-time PCR.

Gene name	Sense/antisense primers-probes	Product length
NogoA	TTGCCTTGCTTAGAATTGCCCTGT (Forward)	162bp
	GCCCATTTCTGTCTGAGGTTCCAA (Reverse)	
*β*-Actin	ACATCCGTAAAGACCTCTATGCC (Forward)	223bp
	TACTCCTGCTTGCTGATCCAC (Reverse)	

**Table 2:** 19 active compounds of AR-RAS.

Mol ID	Molecule Name	OB (%)	DL
MOL000378	7-O-methylisomucronulatol	74.69	0.3
MOL000392	formononetin	69.67	0.21
MOL000433	FA	68.96	0.71
MOL000380	(6aR,11aR)-9,10-dimethoxy-6a,11a-dihydro-6H-benzofurano[3,2-c]chromen-3-ol	64.26	0.42
MOL000211	Mairin	55.38	0.78
MOL000371	3,9-di-O-methylnissolin	53.74	0.48
MOL000239	Jaranol	50.83	0.29
MOL000354	isorhamnetin	49.6	0.31
MOL000439	isomucronulatol-7,2'-di-O-glucosiole	49.28	0.62
MOL000417	Calycosin	47.75	0.24
MOL000098	quercetin	46.43	0.28
MOL000449	Stigmasterol	43.83	0.76
MOL000422	kaempferol	41.88	0.24
MOL000442	1,7-Dihydroxy-3,9-dimethoxy pterocarpene	39.05	0.48
MOL000296	hederagenin	36.91	0.75
MOL000358	beta-sitosterol	36.91	0.75
MOL000379	9,10-dimethoxypterocarpan-3-O-*β*-D-glucoside	36.74	0.92
MOL000033	(3S,8S,9S,10R,13R,14S,17R)-10,13-dimethyl-17-[(2R,5S)-5-propan-2-yloctan-2-yl]-2,3,4,7,8,9,11,12,14,15,16,17-dodecahydro-1H-cyclopenta[a]phenanthren-3-ol	36.23	0.78
MOL000387	Bifendate	31.1	0.67

**Table 3:** Top 10 proteins of TBI-specific proteins according to the degree.

Proteins	Degree
APOE	147
APP	113
GRIN1	68
HTT	63
TNFRSF1A	58
MAPT	57
GRIN2B	53
NTRK1	44
GRIN2A	35
GRIN2D	33

## Data Availability

The data used to support the findings of this study are included in the article and the supplementary information file.

## Abbreviations

AR-RAS: Astragali Radix-Radix Angelica Sinensis
TBI: Traumatic brain injury
TCM: Traditional Chines medicine
DL: Drug likeness
OB: Oral bioavailability
C-T: Compound-target
pC-pT-P: Potential compound-potential target-pathway
TCMSP: Chinese medicine system pharmacology technology platform
TTD: Therapeutic Target Database
OMIM: Online Mendelian Inheritance in Man
HPRD: Human Protein Reference Database
GO: Gene Ontology
KEGG: Kyoto Encyclopedia of Genes and Genomes
GSH: Glutathione synthetase
MDA: Melanoma differentiation-associated protein
GABA: Gamma-aminobutyric acid type B receptor subunit
ACHE: Acetylcholinesterase.

## Supplementary Materials

Supplementary Materials
**Table S1: **Detailed information of all compounds in AR-RAS collected from the TCMSP database and literature, including 87 components of AR and 125 components of RAS.** Table S2: **19 compounds and 226 target proteins of AR-RAS.** Table S3: **Proteins targeted by 17 active compounds of AR-RAS enrichened in 95 signal pathways (P<0.001).** Table S4: **TBI-specific proteins.** Table S5:** 89 signal pathways related to AR-RAS for treating TBI.
