# Supplementary material for: A Network Pharmacology Analysis to Explore the Effect of Astragali Radix-Radix Angelica Sinensis on Traumatic Brain Injury
Source: Biomed Res Int. 2018 Nov 25;2018:3951783. doi: 10.1155/2018/3951783 (PMC6286735; doi:10.1155/2018/3951783)
Supplement: Supplementary Materials — Table S1: Detailed information of all compounds in AR-RAS collected from the TCMSP database and literature, including 87 components of AR and 125 components of RAS. Table S2: 19 compounds and 226 target proteins of AR-RAS. Table S3: Proteins targeted by 17 active compounds of AR-RAS enrichened in 95 signal pathways (P<0.001). Table S4: TBI-specific proteins. Table S5: 89 signal pathways related to AR-RAS for treating TBI. [file 3951783.f1.pdf]

Supplementary Material

A network pharmacology analysis to explore the effect of *Astragali Radix-Radix Angelica Sinensis* on traumatic brain injury

Correspondence: Yang Wang: wangyang_xy87@csu.edu.cn;

Zhihua Xing: XZH_56@163.com.

Supplementary Table S1 Detailed information of all compounds in AR-RAS collected from the TCMSP database and literature, including 87 components of AR and 125 components of RAS.

Herb Name	MOL ID	Molecule Name	OB (%)	DL
Astragali Radix	MOL000114	vanillic acid	35.47	0.04
Astragali Radix	MOL000131	EIC	41.9	0.14
Astragali Radix	MOL000211	Mairin	55.38	0.78
Astragali Radix	MOL001955	Heriguard	11.93	0.33
Astragali Radix	MOL000239	Jaranol	50.83	0.29
Astragali Radix	MOL000251	Rhamnocitrin	12.9	0.27
Astragali Radix	MOL000295	alexandrin	20.63	0.63
Astragali Radix	MOL000296	hederagenin	36.91	0.75
Astragali Radix	MOL000033	(3S,8S,9S,10R,13R,14S,17R)-10,13-dimethyl-17-[(2R,5S)-5-propan-2-yloctan-2-yl]-2,3,4,7,8,9,11,12,14,15,16,17-dodecahydro-1H-cyclopenta[a]phenanthren-3-ol	36.23	0.78
Astragali Radix	MOL000354	isorhamnetin	49.6	0.31
Astragali Radix	MOL000356	lupeol	12.12	0.78
Astragali Radix	MOL000371	3,9-di-O-methylnissolin	53.74	0.48
Astragali Radix	MOL000372	3-Hydroxy-2-picoline	62.47	0.02
Astragali Radix	MOL000373	(2S)-4-methoxy-7-methyl-2-[1-met	5.38	0.81

		hyl-1-[(2S,3R,4S,5S,6R)-3,4,5-trihydroxy-6-methylol-tetrahydropyran-2-yl]oxy-ethyl]-2,3-dihydrofuro[3,2-g]chromen-5-one		
Astragali Radix	MOL000374	5'-hydroxyiso-muronulatol-2',5'-di-O-glucoside	41.72	0.69
Astragali Radix	MOL000375	5'-hydroxyiso-muronulatol-2',5'-di-O-glucoside_qt	3.65	0.8
Astragali Radix	MOL000376	7,2'-dihydroxy-3',4'-dimethoxyisoflavone-7-O-β-D-glucoside	16.16	0.86
Astragali Radix	MOL000377	7-hydroxy-3-(2-hydroxy-3,4-dimethoxy-phenyl)chromone	5.45	0.3
Astragali Radix	MOL000378	7-O-methylisomucronulatol	74.69	0.3
Astragali Radix	MOL000379	9,10-dimethoxypterocarpan-3-O-β-D-glucoside	36.74	0.92
Astragali Radix	MOL000380	(6aR,11aR)-9,10-dimethoxy-6a,11a-dihydro-6H-benzofurano[3,2-c]chromen-3-ol	64.26	0.42
Astragali Radix	MOL000381	13-hydroxy-9,11-octadecadienoic acid	35.6	0.17
Astragali Radix	MOL000382	Arabinose,d	1.87	0.02
Astragali Radix	MOL000383	D-Galacturonic acid, homopolymer	29.75	0.04
Astragali Radix	MOL000384	DL-Glucuronic acid	3.35	0.04
Astragali Radix	MOL005928	isoferulic acid	50.83	0.06
Astragali Radix	MOL000386	Fucopyranose, L-	42.51	0.03
Astragali Radix	MOL000387	Bifendate	31.1	0.67
Astragali Radix	MOL000388	gamma-aminobutyric acid	24.09	0.01
Astragali Radix	MOL000389	FERULIC ACID (CIS)	54.97	0.06
Astragali Radix	MOL000390	daidzein	19.44	0.19

Astragali Radix	MOL000391	Ononin	11.52	0.78
Astragali Radix	MOL000392	formononetin	69.67	0.21
Astragali Radix	MOL000393	Soyasaponin I	2.06	0.05
Astragali Radix	MOL000394	choline	0.47	0.01
Astragali Radix	MOL000395	GGB	54.95	0.03
Astragali Radix	MOL000396	(+)-Syringaresinol	3.29	0.72
Astragali Radix	MOL000397	cis-p-Coumarate	45.98	0.04
Astragali Radix	MOL000398	isoflavanone	109.99	0.3
Astragali Radix	MOL000399	Docosanoate	15.69	0.26
Astragali Radix	MOL000400	Flavaxin	18.18	0.5
Astragali Radix	MOL000401	astragalosideI	46.79	0.11
Astragali Radix	MOL000402	astragalosideI_qt	12.34	0.2
Astragali Radix	MOL000403	astragalosideII	46.06	0.13
Astragali Radix	MOL000404	astragalosideII_qt	11.55	0.25
Astragali Radix	MOL000405	astragalosideIII	31.83	0.1
Astragali Radix	MOL000406	astragalosideIII_qt	5.35	0.32
Astragali Radix	MOL000407	astragalosideIV	22.5	0.15
Astragali Radix	MOL000408	astragalosideIV_qt	7.07	0.32
Astragali Radix	MOL000409	AstragalosideIV	17.74	0.15
Astragali Radix	MOL000410	AstragalosideIV_qt	7.07	0.32
Astragali Radix	MOL000411	Astraisoflavanin	18.37	0.86
Astragali Radix	MOL000412	Mucronulatol	4.22	0.26
Astragali Radix	MOL000413	astrachrysoside A	24.55	0.1
Astragali Radix	MOL000414	Caffeate	54.97	0.05
Astragali Radix	MOL000415	rutin	3.2	0.68
Astragali Radix	MOL000416	Lariciresinol	5.53	0.38
Astragali Radix	MOL000417	Calycosin	47.75	0.24
Astragali Radix	MOL000418	3'-Hydroxy-4'-methoxyisoflavone-7-O-beta-D-glucoside	10.05	0.81

Astragali Radix	MOL000419	astrasieversianin XV	11.19	0.07
Astragali Radix	MOL000420	XLS	51.08	0.02
Astragali Radix	MOL000421	nicotinic acid	47.65	0.02
Astragali Radix	MOL000422	kaempferol	41.88	0.24
Astragali Radix	MOL000423	rhamnocitrin-3-O-glucoside	2.87	0.76
Astragali Radix	MOL000424	RAM	50.5	0.04
Astragali Radix	MOL000425	asernestioside A	11.07	0.03
Astragali Radix	MOL000426	asernestioside A_qt	24.55	0.1
Astragali Radix	MOL000427	asernestioside B	12.54	0.03
Astragali Radix	MOL000428	asernestioside B_qt	14.03	0.09
Astragali Radix	MOL000429	Crystal VI	83.96	0.02
Astragali Radix	MOL000430	betaine	40.92	0.01
Astragali Radix	MOL000431	coumarin	29.17	0.04
Astragali Radix	MOL000432	linolenic acid	45.01	0.15
Astragali Radix	MOL000433	FA	68.96	0.71
Astragali Radix	MOL000434	acetylastragaloside I	43.54	0.09
Astragali Radix	MOL000435	acetylastragaloside I_qt	30.75	0.17
Astragali Radix	MOL000436	(Z)-1-(2,4-dihydroxyphenyl)-3-(4-hydroxyphenyl)prop-2-en-1-one	87.51	0.15
Astragali Radix	MOL000437	Hirsutrin	1.86	0.77
Astragali Radix	MOL000438	(3R)-3-(2-hydroxy-3,4-dimethoxyphenyl)chroman-7-ol	67.67	0.26
Astragali Radix	MOL000439	isomucronulatol-7,2'-di-O-glucosiole	49.28	0.62
Astragali Radix	MOL000440	isomucronulatol-7,2'-di-O-glucosiole_qt	23.42	0.79
Astragali Radix	MOL000441	LUPENONE	11.66	0.78
Astragali Radix	MOL000442	1,7-Dihydroxy-3,9-dimethoxy pterocarpene	39.05	0.48

Astragali Radix	MOL000054	L-	47.64	0.03
Astragali Radix	MOL000061	Prolinum	77.57	0.01
Astragali Radix	MOL000069	palmitic acid	19.3	0.1
Astragali Radix	MOL000098	quercetin	46.43	0.28
Radix Angelica Sinensis	MOL000116	Nonanal	40.28	0.02
Radix Angelica Sinensis	MOL000117	Cymol	27.2	0.02
Radix Angelica Sinensis	MOL000121	Decanal	29.81	0.02
Radix Angelica Sinensis	MOL001212	Loxanol V	14.19	0.05
Radix Angelica Sinensis	MOL001224	Tridecylene	17.69	0.03
Radix Angelica Sinensis	MOL000125	(-)-alpha-Pinene	46.25	0.05
Radix Angelica Sinensis	MOL001273	80-57-9	50.63	0.06
Radix Angelica Sinensis	MOL001302	Usaf hc-1	16.23	0.05
Radix Angelica Sinensis	MOL001306	o-Acetyl-p-cresol	24.96	0.03
Radix Angelica Sinensis	MOL001314	Azelex	16.9	0.04
Radix Angelica Sinensis	MOL001388	(+)-Ledol	16.96	0.12
Radix Angelica Sinensis	MOL001578	Hypnon	48.19	0.02
Radix Angelica Sinensis	MOL000162	beta-Chamigrene	31.99	0.08
Radix Angelica	MOL001640	NON	26.74	0.03

Sinensis				
Radix Angelica Sinensis	MOL001788	adenine	62.81	0.03
Radix Angelica Sinensis	MOL000197	Myrcene	24.96	0.02
Radix Angelica Sinensis	MOL000199	Safrol	45.34	0.05
Radix Angelica Sinensis	MOL000201	p-Ocimene	15.06	0.02
Radix Angelica Sinensis	MOL000202	Moslene	33.02	0.02
Radix Angelica Sinensis	MOL002029	()-Cuparene	38.26	0.07
Radix Angelica Sinensis	MOL002033	cis-Thujopsene	56.43	0.12
Radix Angelica Sinensis	MOL000206	isoeugenol	70.1	0.04
Radix Angelica Sinensis	MOL002098	3-Butylidene-7-hydroxyphthalide	62.68	0.08
Radix Angelica Sinensis	MOL002102	Levistolid A	2.15	0.82
Radix Angelica Sinensis	MOL002110	Allocymene	14.89	0.02
Radix Angelica Sinensis	MOL002111	BdPh	42.44	0.07
Radix Angelica Sinensis	MOL002143	senkyunolide-C	46.8	0.08
Radix Angelica Sinensis	MOL002144	senkyunolide-D	79.13	0.1
Radix Angelica Sinensis	MOL002145	senkyunolide-E	34.4	0.08
Radix Angelica	MOL002180	4-Octanone	19.37	0.01

Sinensis				
Radix Angelica Sinensis	MOL002184	(6R)-6-butylcyclohepta-1,4-diene	31.69	0.02
Radix Angelica Sinensis	MOL002201	cis-ligustilide	51.3	0.07
Radix Angelica Sinensis	MOL000023	Hemo-sol	39.84	0.02
Radix Angelica Sinensis	MOL000232	()-alpha-Terpineol	46.3	0.03
Radix Angelica Sinensis	MOL002480	Methylbutenol	54.58	0.01
Radix Angelica Sinensis	MOL000259	o-Thymol	43.28	0.03
Radix Angelica Sinensis	MOL000270	CHEBI:7	45.2	0.04
Radix Angelica Sinensis	MOL002830	PCR	51.99	0.01
Radix Angelica Sinensis	MOL002972	(4S)-1-methyl-4-(6-methylhepta-1,5-dien-2-yl)cyclohexene	20.3	0.06
Radix Angelica Sinensis	MOL002983	Guasol	51.6	0.02
Radix Angelica Sinensis	MOL002998	IPH	36.05	0.01
Radix Angelica Sinensis	MOL000346	succinic acid	29.62	0.01
Radix Angelica Sinensis	MOL000035	beta-Selinene	24.39	0.08
Radix Angelica Sinensis	MOL003534	CADINENE	17.12	0.08
Radix Angelica Sinensis	MOL000357	Sitogluside	20.63	0.62
Radix Angelica	MOL000358	beta-sitosterol	36.91	0.75

Sinensis				
Radix Angelica Sinensis	MOL003587	Acoradiene	36.73	0.07
Radix Angelica Sinensis	MOL000360	FER	39.56	0.06
Radix Angelica Sinensis	MOL000383	D-Galacturonic acid, homopolymer	29.75	0.04
Radix Angelica Sinensis	MOL000389	FERULIC ACID (CIS)	54.97	0.06
Radix Angelica Sinensis	MOL000040	Scopoletol	27.77	0.08
Radix Angelica Sinensis	MOL000421	nicotinic acid	47.65	0.02
Radix Angelica Sinensis	MOL004474	Maruzen M	48.44	0.02
Radix Angelica Sinensis	MOL004479	o-cresol	62.45	0.02
Radix Angelica Sinensis	MOL004613	6,7,3',8'-diligustilide	9.83	0.7
Radix Angelica Sinensis	MOL004723	beta-Terpinene	42.29	0.02
Radix Angelica Sinensis	MOL004734	Butal	68.66	0
Radix Angelica Sinensis	MOL000478	Eucarvone	53.14	0.03
Radix Angelica Sinensis	MOL000479	Farnesene	17.42	0.05
Radix Angelica Sinensis	MOL004791	Ethol	13.32	0.08
Radix Angelica Sinensis	MOL000489	(1S,4aR,8aR)-1-isopropyl-7-methyl-4-methylene-2,3,4a,5,6,8a-hexahydro-1H-naphthalene	19.8	0.08

Radix Angelica Sinensis	MOL005125	ANN	29.69	0.03
Radix Angelica Sinensis	MOL005449	h-Met-h	70.87	0.01
Radix Angelica Sinensis	MOL000449	Stigmasterol	43.83	0.76
Radix Angelica Sinensis	MOL005589	3,4-DIMETHYLBENZALDEHYDE	39.99	0.02
Radix Angelica Sinensis	MOL005590	ETHYLBENZALDEHYDE	40.95	0.02
Radix Angelica Sinensis	MOL005608	TMHYDROP	54.42	0.03
Radix Angelica Sinensis	MOL000635	vanillin	52	0.03
Radix Angelica Sinensis	MOL006440	bicycloelemene	20.89	0.08
Radix Angelica Sinensis	MOL006869	WLN: QR CQ DV1	36.49	0.03
Radix Angelica Sinensis	MOL000069	palmitic acid	19.3	0.1
Radix Angelica Sinensis	MOL000705	WLN: VH6	19.59	0.01
Radix Angelica Sinensis	MOL007745	WLN: QVR BVQ	17.74	0.04
Radix Angelica Sinensis	MOL008244	(1R,2S,4R)-1-ethyl-1-methyl-2,4-bis(1-methylethyl)cyclohexane	15.01	0.06
Radix Angelica Sinensis	MOL008245	DODECENE	17.74	0.02
Radix Angelica Sinensis	MOL008246	phosphatdic acid	19.32	0.05
Radix Angelica Sinensis	MOL008247	L-beta,gamma-Dimyristoyl-alpha-c	20.69	0.47

		ephalin		
Radix Angelica Sinensis	MOL008248	phosphatidylinositol	4.63	0.29
Radix Angelica Sinensis	MOL008249	phosphatidylinositol_qt	12.66	0.05
Radix Angelica Sinensis	MOL008250	ESEN	47.31	0.04
Radix Angelica Sinensis	MOL008251	sedanolide	62.46	0.07
Radix Angelica Sinensis	MOL008252	senkyunolide	68.28	0.07
Radix Angelica Sinensis	MOL008253	sphingomyelin	0.31	0.51
Radix Angelica Sinensis	MOL008254	Isotetandrine	10.42	0.1
Radix Angelica Sinensis	MOL008255	α-acoradiene	40.98	0.07
Radix Angelica Sinensis	MOL008256	InChI=1/C15H24/c1-10-7-8-15-9-12(10)14(3,4)13(15)6-5-11(15)2/h7,11-13H,5-6,8-9H2,1-4H	55.56	0.1
Radix Angelica Sinensis	MOL008257	α-copaene	29.33	0.12
Radix Angelica Sinensis	MOL008258	(1R,4R,5S)-4-isopropenyl-1,8-dimethylspiro[4.5]dec-8-ene	40.65	0.07
Radix Angelica Sinensis	MOL008259	2,6-di(phenyl)thiopyran-4-thione	69.13	0.15
Radix Angelica Sinensis	MOL008260	o-Xylenol	53.13	0.02
Radix Angelica Sinensis	MOL008261	2,4,6-trimethyl-Octane	29.14	0.02
Radix Angelica Sinensis	MOL008262	Mesitaldehyde	37.8	0.03

Radix Angelica Sinensis	MOL008263	Isoxylaldehyde	38.85	0.02
Radix Angelica Sinensis	MOL008264	(E)-octadec-3-ene	19.5	0.09
Radix Angelica Sinensis	MOL008265	2-valerylbenzoic acid	78.26	0.06
Radix Angelica Sinensis	MOL008266	(Z)-2-Hexenyl hexanoate	19.39	0.04
Radix Angelica Sinensis	MOL008267	2-Methylhexadecanoic acid	20.23	0.11
Radix Angelica Sinensis	MOL008268	cis-Isoeugenol	20.73	0.04
Radix Angelica Sinensis	MOL008269	2-methyl-5-decanone	20.4	0.11
Radix Angelica Sinensis	MOL008270	2-methyldodecan-5-one	13.16	0.04
Radix Angelica Sinensis	MOL008271	3,7-dimethyl-nonane	16.97	0.02
Radix Angelica Sinensis	MOL008272	1,1,5-trimethyl-2-formylcyclohexa-2,5-diene-4-one	48.94	0.04
Radix Angelica Sinensis	MOL008273	4-Methyl-6-hepten-3-one	78.38	0.01
Radix Angelica Sinensis	MOL008274	6-Ethylresorcinol	46.45	0.03
Radix Angelica Sinensis	MOL008275	5-Indolol	63.14	0.03
Radix Angelica Sinensis	MOL008276	Undecanol-6	25.77	0.02
Radix Angelica Sinensis	MOL008277	7,10-PENTADECADIYNOIC ACID	41.5	0.09
Radix Angelica Sinensis	MOL008278	4-chloro-N-[1-methyl-5-[[1-methyl -5-[[1-methyl-5-(2-morpholinoethyl	7.18	0.31

		carbamoyl)pyrrol-3-yl]carbamoyl]pyrrol-3-yl]carbamoyl]pyrrol-3-yl]-5-[2-(2-pyridyl)ethylamino]isothiazole-3-carboxamide		
Radix Angelica Sinensis	MOL008279	Amyl ketone	18.65	0.02
Radix Angelica Sinensis	MOL008280	Isoamylbenzene	35.69	0.03
Radix Angelica Sinensis	MOL008281	(Z)-2-[[(Z)-2-methylbut-2-enoyl]oxymethyl]but-2-enoic acid	77.1	0.04
Radix Angelica Sinensis	MOL008282	Tropone	47.41	0.01
Radix Angelica Sinensis	MOL008283	aromadendrene	18.21	0.1
Radix Angelica Sinensis	MOL008284	BUA	21.62	0
Radix Angelica Sinensis	MOL008285	(3S)-3-butyl-3H-isobenzofuran-1-one	55.05	0.07
Radix Angelica Sinensis	MOL008286	()-Camphoric acid	99.13	0.07
Radix Angelica Sinensis	MOL008287	(3E)-3-butylidene-7-hydroxy-2-benzofuran-1-one	42.17	0.08
Radix Angelica Sinensis	MOL008288	Coniferyl ferulate	4.54	0.39
Radix Angelica Sinensis	MOL008290	lecithin	0.31	0.4
Radix Angelica Sinensis	MOL008291	2,4-Xylylaldehyde	39.33	0.03
Radix Angelica Sinensis	MOL008292	m-Ethylphenol	51.3	0.02
Radix Angelica	MOL008293	1,5,5,6-tetramethyl-1,3-Cyclohexad	39.22	0.03

Sinensis		iene		
Radix Angelica Sinensis	MOL000885	Dodekan	17.74	0.02
Radix Angelica Sinensis	MOL000967	bergamotene	28.51	0.09
Radix Angelica Sinensis	MOL000974	cuminal	38.29	0.03

Supplementary Table S2 19 compounds and 226 target proteins of AR-RAS.

Ingredients	Related Targets	Gene names
7-O-Methylisomucronulatol	Nitric oxide synthase, inducible	NOS2
7-O-Methylisomucronulatol	Prostaglandin G/H synthase 1	PTGS1
7-O-Methylisomucronulatol	Dopamine D1 receptor	DRD1
7-O-Methylisomucronulatol	Muscarinic acetylcholine receptor M3	CHRM3
7-O-Methylisomucronulatol	Thrombin	F2
7-O-Methylisomucronulatol	Potassium voltage-gated channel subfamily H member 2	KCNH2
7-O-Methylisomucronulatol	Muscarinic acetylcholine receptor M1	CHRM1
7-O-Methylisomucronulatol	Estrogen receptor	ESR1
7-O-Methylisomucronulatol	Androgen receptor	AR
7-O-Methylisomucronulatol	Beta-1 adrenergic receptor	ADRB1
7-O-Methylisomucronulatol	Sodium channel protein type 5 subunit alpha	SCN5A
7-O-Methylisomucronulatol	Peroxisome proliferator activated receptor gamma	PPARG
7-O-Methylisomucronulatol	Coagulation factor Xa	F10
7-O-Methylisomucronulatol	Muscarinic acetylcholine receptor M5	CHRM5

7-O-Methylisomucronulatol	Prostaglandin G/H synthase 2	PTGS2
7-O-Methylisomucronulatol	Nitric-oxide synthase, endothelial	NOS3
7-O-Methylisomucronulatol	Alpha-2C adrenergic receptor	ADRA2C
7-O-Methylisomucronulatol	Muscarinic acetylcholine receptor M4	CHRM4
7-O-Methylisomucronulatol	Retinoic acid receptor RXR-alpha	RXRA
7-O-Methylisomucronulatol	Delta-type opioid receptor	OPRD1
7-O-Methylisomucronulatol	CGMP-inhibited 3',5'-cyclic phosphodiesterase A	PDE3A
7-O-Methylisomucronulatol	5-hydroxytryptamine 2A receptor	HTR2A
7-O-Methylisomucronulatol	Alpha-1A adrenergic receptor	ADRA1A
7-O-Methylisomucronulatol	Muscarinic acetylcholine receptor M2	CHRM2
7-O-Methylisomucronulatol	Alpha-1B adrenergic receptor	ADRA1B
7-O-Methylisomucronulatol	Sodium-dependent dopamine transporter	SLC6A3
7-O-Methylisomucronulatol	Beta-2 adrenergic receptor	ADRB2
7-O-Methylisomucronulatol	Alpha-1D adrenergic receptor	ADRA1D
7-O-Methylisomucronulatol	Sodium-dependent serotonin transporter	SLC6A4
7-O-Methylisomucronulatol	Estrogen receptor beta	ESRRB
7-O-Methylisomucronulatol	Gamma-aminobutyric acid receptor subunit alpha-1	GABRA1
7-O-Methylisomucronulatol	Dipeptidyl peptidase IV	DPP4
7-O-Methylisomucronulatol	Mitogen-activated protein kinase 14	MAPK14
7-O-Methylisomucronulatol	Glycogen synthase kinase-3 beta	GSK3B
7-O-Methylisomucronulatol	Heat shock protein HSP 90	HSP 90

7-O-Methylisomucronulatol	Cell division protein kinase 2	CDK2
7-O-Methylisomucronulatol	Serine/threonine-protein kinase Chk1	CHEK1
7-O-Methylisomucronulatol	mRNA of PKA Catalytic Subunit C-alpha	PRKACA
7-O-Methylisomucronulatol	Retinoic acid receptor RXR-beta	RXRB
7-O-Methylisomucronulatol	Trypsin-1	PRSS1
7-O-Methylisomucronulatol	Proto-oncogene serine/threonine-protein kinase Pim-1	PIM1
7-O-Methylisomucronulatol	Cyclin-A2	CCNA2
7-O-Methylisomucronulatol	Nuclear receptor coactivator 2	NCOA2
7-O-Methylisomucronulatol	Calcium-activated potassium channel subunit alpha 1	KCNMA1
7-O-Methylisomucronulatol	Calmodulin	CALM
Formononetin	Nitric oxide synthase, inducible	NOS2
Formononetin	Prostaglandin G/H synthase 1	PTGS1
Formononetin	Muscarinic acetylcholine receptor M1	CHRM1
Formononetin	Estrogen receptor	ESR2
Formononetin	Androgen receptor	AR
Formononetin	Peroxisome proliferator activated receptor gamma	PPARG
Formononetin	Prostaglandin G/H synthase 2	PTGS2
Formononetin	Retinoic acid receptor RXR-alpha	RXRA
Formononetin	CGMP-inhibited 3',5'-cyclic phosphodiesterase A	PDE3A
Formononetin	Alpha-1A adrenergic receptor	ADRA1A

Formononetin	Sodium-dependent dopamine transporter	SLC6A3
Formononetin	Beta-2 adrenergic receptor	ADRB2
Formononetin	Sodium-dependent serotonin transporter	SLC6A4
Formononetin	Estrogen receptor beta	MAOB
Formononetin	Dipeptidyl peptidase IV	DPP4
Formononetin	Mitogen-activated protein kinase 14	MAPK14
Formononetin	Glycogen synthase kinase-3 beta	GSK3B
Formononetin	Heat shock protein HSP 90	HSP90
Formononetin	Cell division protein kinase 2	CDK2
Formononetin	Amine oxidase [flavin-containing] B	PKIA
Formononetin	Serine/threonine-protein kinase Chk1	CHEK1
Formononetin	mRNA of PKA Catalytic Subunit C-alpha	PRKACA
Formononetin	Trypsin-1	PRSS1
Formononetin	Proto-oncogene serine/threonine-protein kinase Pim-1	PIM1
Formononetin	Cyclin-A2	CCNA2
Formononetin	Calmodulin	CALM
Formononetin	cAMP-dependent protein kinase inhibitor alpha	ACHE
Formononetin	Thrombin	F2
Formononetin	Nitric-oxide synthase, endothelial	NOS3
Formononetin	Acetylcholinesterase	LACTB2

Formononetin	Beta-lactamase	JUN
Formononetin	Transcription factor AP-1	IL4
Formononetin	Peroxisome proliferator-activated receptor gamma	PPARG
Formononetin	Interleukin-4	SIRT1
Formononetin	NAD-dependent deacetylase sirtuin-1	ATP5F1B
Formononetin	ATP synthase subunit beta, mitochondrial	MT-ND6
Formononetin	NADH-ubiquinone oxidoreductase chain 6	HSD3B2
Formononetin	3 beta-hydroxysteroid dehydrogenase/Delta 5-->4-isomerase type 2	HSD3B1
Formononetin	3 beta-hydroxysteroid dehydrogenase/Delta 5-->4-isomerase type 1	HTR3A
FA	Cell division protein kinase 2	CDK2
FA	Thrombin	F2
FA	Glycogen synthase kinase-3 beta	GSK3B
(6aR,11aR)-9,10-dimethoxy-6a,11a-dihydro-6H-benzofurano[3,2-c]chromen-3-ol	Nitric oxide synthase, inducible	NOS2
(6aR,11aR)-9,10-dimethoxy-6a,11a-dihydro-6H-benzofurano[3,2-c]chromen-3-ol	Prostaglandin G/H synthase 1	PTGS1
(6aR,11aR)-9,10-dimethoxy-6a,11a-dihydro-6H-benzofurano[3,2-c]chromen-3-ol	Muscarinic acetylcholine receptor M3	CHRNA7

(6aR,11aR)-9,10-dimethoxy-6a,11a-dihydro-6H-benzofurano[3,2-c]chromen-3-ol	Thrombin	F2
(6aR,11aR)-9,10-dimethoxy-6a,11a-dihydro-6H-benzofurano[3,2-c]chromen-3-ol	Muscarinic acetylcholine receptor M1	CHRM1
(6aR,11aR)-9,10-dimethoxy-6a,11a-dihydro-6H-benzofurano[3,2-c]chromen-3-ol	Estrogen receptor	ESR1
(6aR,11aR)-9,10-dimethoxy-6a,11a-dihydro-6H-benzofurano[3,2-c]chromen-3-ol	Sodium channel protein type 5 subunit alpha	SCN5A
(6aR,11aR)-9,10-dimethoxy-6a,11a-dihydro-6H-benzofurano[3,2-c]chromen-3-ol	Prostaglandin G/H synthase 2	PTGS2
(6aR,11aR)-9,10-dimethoxy-6a,11a-dihydro-6H-benzofurano[3,2-c]chromen-3-ol	5-hydroxytryptamine receptor 3A	NCOA1
(6aR,11aR)-9,10-dimethoxy-6a,11a-dihydro-6H-benzofurano[3,2-c]chromen-3-ol	Retinoic acid receptor RXR-alpha	PGR
(6aR,11aR)-9,10-dimethoxy-6a,11a-dihydro-6H-benzofurano[3,2-c]chromen-3-ol	Acetylcholinesterase	LACTB2
(6aR,11aR)-9,10-dimethoxy-6a,11a-dihydro-6H-benzofurano[3,2-c]chromen-3-ol	Alpha-1B adrenergic receptor	ADRA1B
(6aR,11aR)-9,10-dimethoxy-6a,11a-dihydro-6H-benzofurano[3,	Beta-2 adrenergic receptor	ADRB2

2-c]chromen-3-ol		
(6aR,11aR)-9,10-dimethoxy-6a, 11a-dihydro-6H-benzofurano[3, 2-c]chromen-3-ol	Alpha-1D adrenergic receptor	ADRA1D
(6aR,11aR)-9,10-dimethoxy-6a, 11a-dihydro-6H-benzofurano[3, 2-c]chromen-3-ol	Gamma-aminobutyric acid receptor subunit alpha-1	GABRA1
(6aR,11aR)-9,10-dimethoxy-6a, 11a-dihydro-6H-benzofurano[3, 2-c]chromen-3-ol	Heat shock protein HSP 90	HSP 90
(6aR,11aR)-9,10-dimethoxy-6a, 11a-dihydro-6H-benzofurano[3, 2-c]chromen-3-ol	Neuronal acetylcholine receptor protein, alpha-7 chain	OPRM1
(6aR,11aR)-9,10-dimethoxy-6a, 11a-dihydro-6H-benzofurano[3, 2-c]chromen-3-ol	Trypsin-1	PRSS1
(6aR,11aR)-9,10-dimethoxy-6a, 11a-dihydro-6H-benzofurano[3, 2-c]chromen-3-ol	Nuclear receptor coactivator 2	NCOA2
(6aR,11aR)-9,10-dimethoxy-6a, 11a-dihydro-6H-benzofurano[3, 2-c]chromen-3-ol	Nuclear receptor coactivator 1	NCOA1
(6aR,11aR)-9,10-dimethoxy-6a, 11a-dihydro-6H-benzofurano[3, 2-c]chromen-3-ol	Calmodulin	CALM
(6aR,11aR)-9,10-dimethoxy-6a, 11a-dihydro-6H-benzofurano[3, 2-c]chromen-3-ol	Muscarinic acetylcholine receptor M4	CHRM4
Mairin	Progesterone receptor	PGR

3,9-Di-O-methylnissolin	Nitric oxide synthase, inducible	NOS2
3,9-Di-O-methylnissolin	Prostaglandin G/H synthase 1	PTGS1
3,9-Di-O-methylnissolin	Muscarinic acetylcholine receptor M3	CHRNA7
3,9-Di-O-methylnissolin	Thrombin	F2
3,9-Di-O-methylnissolin	Muscarinic acetylcholine receptor M1	CHRM1
3,9-Di-O-methylnissolin	Estrogen receptor	ESR1
3,9-Di-O-methylnissolin	Beta-1 adrenergic receptor	ADRB1
3,9-Di-O-methylnissolin	Sodium channel protein type 5 subunit alpha	SCN5A
3,9-Di-O-methylnissolin	Prostaglandin G/H synthase 2	PTGS2
3,9-Di-O-methylnissolin	Nitric-oxide synthase, endothelial	NOS3
3,9-Di-O-methylnissolin	5-hydroxytryptamine receptor 3A	HTR3A
3,9-Di-O-methylnissolin	Alpha-2C adrenergic receptor	ADRA2C
3,9-Di-O-methylnissolin	Retinoic acid receptor RXR-alpha	RXRA
3,9-Di-O-methylnissolin	Acetylcholinesterase	LACTB2
3,9-Di-O-methylnissolin	CGMP-inhibited 3',5'-cyclic phosphodiesterase A	PDE3A
3,9-Di-O-methylnissolin	Alpha-1B adrenergic receptor	ADRA1B
3,9-Di-O-methylnissolin	Beta-2 adrenergic receptor	ADRB2
3,9-Di-O-methylnissolin	Alpha-1D adrenergic receptor	ADRA1D
3,9-Di-O-methylnissolin	Mu-type opioid receptor	OPRM1
3,9-Di-O-methylnissolin	Gamma-aminobutyric acid receptor subunit alpha-1	GABRA1
3,9-Di-O-methylnissolin	Trypsin-1	PRSS1
3,9-Di-O-methylnissolin	Nuclear receptor coactivator 2	NCOA2
3,9-Di-O-methylnissolin	Calmodulin	CALM

Jaranol	Nitric oxide synthase, inducible	NOS2
Jaranol	Prostaglandin G/H synthase 1	PTGS1
Jaranol	Androgen receptor	AR
Jaranol	Sodium channel protein type 5 subunit alpha	SCN5A
Jaranol	Prostaglandin G/H synthase 2	PTGS2
Jaranol	Estrogen receptor beta	MAOB
Jaranol	Dipeptidyl peptidase IV	DPP4
Jaranol	Heat shock protein HSP 90	HSP 90
Jaranol	Cell division protein kinase 2	CDK2
Jaranol	Serine/threonine-protein kinase Chk1	CHEK1
Jaranol	Trypsin-1	PRSS1
Jaranol	Nuclear receptor coactivator 2	NCOA2
Jaranol	Calmodulin	CALM
Isorhamnetin	Nitric oxide synthase, inducible	NOS2
Isorhamnetin	Prostaglandin G/H synthase 1	PTGS1
Isorhamnetin	Estrogen receptor	ESR1
Isorhamnetin	Androgen receptor	AR
Isorhamnetin	Peroxisome proliferator activated receptor gamma	PPARG
Isorhamnetin	Prostaglandin G/H synthase 2	PTGS2
Isorhamnetin	mRNA of Protein-tyrosine phosphatase, non-receptor type 1	PTPN1
Isorhamnetin	Estrogen receptor beta	MAOB
Isorhamnetin	Dipeptidyl peptidase IV	DPP4
Isorhamnetin	Mitogen-activated protein kinase 14	MAPK14
Isorhamnetin	Glycogen synthase kinase-3 beta	GSK3B

Isorhamnetin	Heat shock protein HSP 90	HSP 90
Isorhamnetin	Cell division protein kinase 2	CDK2
Isorhamnetin	Phosphatidylinositol-4,5-bisphosphate 3-kinase catalytic subunit, gamma isoform	PIK3CG
Isorhamnetin	mRNA of PKA Catalytic Subunit C-alpha	PRKACA
Isorhamnetin	Trypsin-1	PRSS1
Isorhamnetin	Proto-oncogene serine/threonine-protein kinase Pim-1	PIM1
Isorhamnetin	Cyclin-A2	CCNA2
Isorhamnetin	Nuclear receptor coactivator 2	NCOA2
Isorhamnetin	Calmodulin	CALM
Isorhamnetin	Glycogen phosphorylase, muscle form	PYGM
Isorhamnetin	Peroxisome proliferator activated receptor delta	PPARD
Isorhamnetin	Serine/threonine-protein kinase Chk1	CHEK1
Isorhamnetin	Aldose reductase	AKR1B1
Isorhamnetin	Nuclear receptor coactivator 1	NCOA1
Isorhamnetin	Coagulation factor VII	F7
Isorhamnetin	Thrombin	F2
Isorhamnetin	Nitric-oxide synthase, endothelial	NOS3
Isorhamnetin	Acetylcholinesterase	LACTB2
Isorhamnetin	Gamma-aminobutyric acid receptor subunit alpha-1	GABRA1
Isorhamnetin	Amine oxidase	PKIA

	[flavin-containing] B	
Isorhamnetin	Glutamate receptor 2	GRIA2
Isorhamnetin	Cytochrome P450-cam	CAMC
Isorhamnetin	Transcription factor p65	RELA
Isorhamnetin	Xanthine dehydrogenase/oxidase	XDH
Isorhamnetin	Neutrophil cytosol factor 1	NCOA1
Isorhamnetin	Oxidized low-density lipoprotein receptor 1	OLR1
isomucronulatol-7,2'-di-O-glucosiole	DNA topoisomerase II	TOP2
Calycosin	Nitric oxide synthase, inducible	NOS2
Calycosin	Prostaglandin G/H synthase 1	PTGS1
Calycosin	Estrogen receptor	ESR1
Calycosin	Androgen receptor	AR
Calycosin	Peroxisome proliferator activated receptor gamma	PPARG
Calycosin	Prostaglandin G/H synthase 2	PTGS2
Calycosin	Retinoic acid receptor RXR-alpha	RXRA
Calycosin	CGMP-inhibited 3',5'-cyclic phosphodiesterase A	PDE3A
Calycosin	Estrogen receptor beta	MAOB
Calycosin	Dipeptidyl peptidase IV	DPP4
Calycosin	Mitogen-activated protein kinase 14	MAPK14
Calycosin	Glycogen synthase kinase-3 beta	GSK3B
Calycosin	Heat shock protein HSP 90	HSP 90
Calycosin	Cell division protein kinase 2	CDK2
Calycosin	Serine/threonine-protein kinase	CHEK1

	Chk1	
Calycosin	mRNA of PKA Catalytic Subunit C-alpha	PRKACA
Calycosin	Trypsin-1	PRSS1
Calycosin	Proto-oncogene serine/threonine-protein kinase Pim-1	PIM1
Calycosin	Cyclin-A2	CCNA2
Calycosin	Nuclear receptor coactivator 2	NCOA2
Calycosin	Calmodulin	CALM
Calycosin	Beta-2 adrenergic receptor	ADRB2
quercetin	Prostaglandin G/H synthase 1	PTGS1
quercetin	Androgen receptor	AR
quercetin	Peroxisome proliferator activated receptor gamma	PPARG
quercetin	Prostaglandin G/H synthase 2	PTGS2
quercetin	Heat shock protein HSP 90	HSP 90
quercetin	Phosphatidylinositol-4,5-bisphosphate 3-kinase catalytic subunit, gamma isoform	PIK3CG
quercetin	Nuclear receptor coactivator 2	NCOA2
quercetin	Dipeptidyl peptidase IV	DPP4
quercetin	Aldose reductase	AKR1B1
quercetin	Trypsin-1	PRSS1
quercetin	DNA topoisomerase II	TOP2
quercetin	Thrombin	F2
quercetin	Potassium voltage-gated channel subfamily H member 2	KCNH2
quercetin	Sodium channel protein type 5	SCN5A

	subunit alpha	
quercetin	Coagulation factor Xa	F10
quercetin	Beta-2 adrenergic receptor	ADRB2
quercetin	Stromelysin-1	MMP3
quercetin	mRNA of PKA Catalytic Subunit C-alpha	PRKACA
quercetin	Coagulation factor VII	F7
quercetin	Nitric-oxide synthase, endothelial	NOS3
quercetin	Retinoic acid receptor RXR-alpha	RXRA
quercetin	Acetylcholinesterase	LACTB2
quercetin	Gamma-aminobutyric acid receptor subunit alpha-1	GABRA1
quercetin	Amine oxidase [flavin-containing] B	PKIA
quercetin	Transcription factor p65	RELA
quercetin	Epidermal growth factor receptor	EGFR
quercetin	RAC-alpha serine/threonine-protein kinase	AKT1
quercetin	Vascular endothelial growth factor A	VEGFA
quercetin	G1/S-specific cyclin-D1	CCND1
quercetin	Apoptosis regulator Bcl-2	BCL2
quercetin	Bcl-2-like protein 1	BCL2L1
quercetin	Proto-oncogene c-Fos	FOS
quercetin	Cyclin-dependent kinase inhibitor 1	CDKN1
quercetin	Eukaryotic translation initiation factor 6	EIF6

quercetin	Apoptosis regulator BAX	BAX
quercetin	Caspase-9	CASP9
quercetin	Urokinase-type plasminogen activator	PLAU
quercetin	72 kDa type IV collagenase	MMP2
quercetin	Matrix metalloproteinase-9	MMP9
quercetin	Mitogen-activated protein kinase 1	MAPK1
quercetin	Interleukin-10	IL10
quercetin	Pro-epidermal growth factor	EGF
quercetin	Retinoblastoma-associated protein	RB1
quercetin	Tumor necrosis factor	TNF
quercetin	Transcription factor AP-1	IL4
quercetin	Interleukin-6	IL6
quercetin	Cyclin-dependent kinase inhibitor 2A, isoforms 1/2/3	CDKN2A
quercetin	Activator of 90 kDa heat shock protein ATPase homolog 1	AHSA1
quercetin	Caspase-3	CASP3
quercetin	Cellular tumor antigen p53	TP53
quercetin	ETS domain-containing protein Elk-1	ELK1
quercetin	NF-kappa-B inhibitor alpha	NFKBIA
quercetin	NADPH--cytochrome P450 reductase	POR
quercetin	Ornithine decarboxylase	ODC1
quercetin	Xanthine dehydrogenase/oxidase	XDH
quercetin	Caspase-8	CASP8

quercetin	DNA topoisomerase 1	TOP1
quercetin	RAF proto-oncogene serine/threonine-protein kinase	RAF1
quercetin	Superoxide dismutase [Cu-Zn]	SOD1
quercetin	Protein kinase C alpha type	PRKCA
quercetin	Interstitial collagenase	MMP1
quercetin	Hypoxia-inducible factor 1-alpha	HIF1A
quercetin	Signal transducer and activator of transcription 1-alpha/beta	STAT1
quercetin	Protein CBFA2T1	RUNX1T1
quercetin	Probable E3 ubiquitin-protein ligase HERC5	HERC5
quercetin	Cell division control protein 2 homolog	CDK1
quercetin	78 kDa glucose-regulated protein	HSPA5
quercetin	Receptor tyrosine-protein kinase erbB-2	ERBB2
quercetin	Peroxisome proliferator-activated receptor gamma	PPARG
quercetin	Acetyl-CoA carboxylase 1	ACACA
quercetin	Heme oxygenase 1	HMOX1
quercetin	Cytochrome P450 3A4	CYP3A4
quercetin	Cytochrome P450 1A2	CYP1A2
quercetin	Caveolin-1	CAV1
quercetin	Myc proto-oncogene protein	MYC
quercetin	Tissue factor	F3
quercetin	Gap junction alpha-1 protein	GJA1
quercetin	Cytochrome P450 1A1	CYP1A1
quercetin	Intercellular adhesion molecule 1	ICAM1

quercetin	Interleukin-1 beta	IL1B
quercetin	C-C motif chemokine 2	CCL2
quercetin	E-selectin	SELE
quercetin	Vascular cell adhesion protein 1	VCAM1
quercetin	Prostaglandin E2 receptor EP3 subtype	PTGER3
quercetin	Interleukin-8	CXCL8
quercetin	Protein kinase C beta type	PRKCB
quercetin	Baculoviral IAP repeat-containing protein 5	BIRC5
quercetin	Dual oxidase 2	DUOX2
quercetin	Nitric oxide synthase, endothelial	NOS3
quercetin	Heat shock protein beta-1	HSPB1
quercetin	Transforming growth factor beta-1	TGFB1
quercetin	Estrogen sulfotransferase	SULT1E1
quercetin	Maltase-glucoamylase, intestinal	MGAM
quercetin	Interleukin-2	IL2
quercetin	Nuclear receptor subfamily 1 group I member 2	NR2C2
quercetin	Cytochrome P450 1B1	CYP1B1
quercetin	G2/mitotic-specific cyclin-B1	CCNB1
quercetin	Tissue-type plasminogen activator	PLAT
quercetin	Thrombomodulin	THBD
quercetin	Plasminogen activator inhibitor 1	SERPINE1
quercetin	Collagen alpha-1(I) chain	COL1A1
quercetin	Interferon gamma	IFNG
quercetin	Arachidonate 5-lipoxygenase	ALOX5

quercetin	Phosphatidylinositol-3,4,5-trisphosphate 3-phosphatase and dual-specificity protein phosphatase PTEN	PTEN
quercetin	Interleukin-1 alpha	IL1A
quercetin	Myeloperoxidase	MPO
quercetin	DNA topoisomerase 2-alpha	TOP2A
quercetin	Neutrophil cytosol factor 1	NCF1
quercetin	ATP-binding cassette sub-family G member 2	ABCG2
quercetin	Hyaluronan synthase 2	HAS2
quercetin	Glutathione S-transferase P	GSTP1
quercetin	Nuclear factor erythroid 2-related factor 2	NFE2L2
quercetin	NAD(P)H dehydrogenase [quinone] 1	NQO1
quercetin	Poly [ADP-ribose] polymerase 1	PARP1
quercetin	Aryl hydrocarbon receptor	AHR
quercetin	26S proteasome non-ATPase regulatory subunit 3	PSMD3
quercetin	Solute carrier family 2, facilitated glucose transporter member 4	SLC2A4
quercetin	Collagen alpha-1(III) chain	COL3A1
quercetin	DNA gyrase subunit B	GYRB
quercetin	C-X-C motif chemokine 11	CXCL11
quercetin	C-X-C motif chemokine 2	CXCL2
quercetin	DDB1- and CUL4-associated factor 5	DCAF5
quercetin	Nuclear receptor subfamily 1	NR1I3

	group I member 3	
quercetin	Serine/threonine-protein kinase Chk2	CHEK2
quercetin	Insulin receptor	INSR
quercetin	Claudin-4	CLDN4
quercetin	Peroxisome proliferator-activated receptor alpha	PPARA
quercetin	Peroxisome proliferator-activated receptor delta	PPARD
quercetin	Heat shock factor protein 1	HSF1
quercetin	C-reactive protein	CRP
quercetin	C-X-C motif chemokine 10	CXCL10
quercetin	Inhibitor of nuclear factor kappa-B kinase subunit alpha	CHUK
quercetin	Osteopontin	SPP1
quercetin	Runt-related transcription factor 2	RUNX2
quercetin	Ras association domain-containing protein 1	RASSF1
quercetin	Transcription factor E2F1	E2F1
quercetin	Transcription factor E2F2	E2F2
quercetin	Prostatic acid phosphatase	ACPP
quercetin	Cathepsin D	CTSD
quercetin	Insulin-like growth factor-binding protein 3	IGFBP3
quercetin	Insulin-like growth factor II	IGF2
quercetin	CD40 ligand	CD40LG
quercetin	Interferon regulatory factor 1	IRF1
quercetin	Receptor tyrosine-protein kinase	ERBB3

	erbB-3	
quercetin	Serum paraoxonase/arylesterase 1	PON1
quercetin	Type I iodothyronine deiodinase	DIO1
quercetin	Procollagen C-endopeptidase enhancer 1	PCOLCE
quercetin	Puromycin-sensitive aminopeptidase	NPEPPS
quercetin	Hexokinase-2	HK2
quercetin	Homeobox protein Nkx-3.1	NKX3-1
quercetin	Ras GTPase-activating protein 1	RASA1
quercetin	Peroxidase C1A	PRXC1A
quercetin	Glutathione S-transferase Mu 1	GSTM1
quercetin	Glutathione S-transferase Mu 2	GSTM2
kaempferol	Nitric oxide synthase, inducible	NOS2
kaempferol	Prostaglandin G/H synthase 1	PTGS1
kaempferol	Androgen receptor	AR
kaempferol	Peroxisome proliferator activated receptor gamma	PPARG
kaempferol	Prostaglandin G/H synthase 2	PTGS2
kaempferol	Heat shock protein HSP 90	HSP 90
kaempferol	Phosphatidylinositol-4,5-bisphosphate 3-kinase catalytic subunit, gamma isoform	PIK3CG
kaempferol	mRNA of PKA Catalytic Subunit C-alpha	PRKACA
kaempferol	Nuclear receptor coactivator 2	NCOA2
kaempferol	Dipeptidyl peptidase IV	DPP4
kaempferol	Trypsin-1	PRSS1

kaempferol	Progesterone receptor	PGR
kaempferol	Thrombin	F2
kaempferol	Muscarinic acetylcholine receptor M1	CHRM1
kaempferol	Nitric-oxide synthase, endothelial	NOS3
kaempferol	Gamma-aminobutyric-acid receptor alpha-2 subunit	GABRA2
kaempferol	Acetylcholinesterase	LACTB2
kaempferol	Sodium-dependent noradrenaline transporter	SLC6A2
kaempferol	Muscarinic acetylcholine receptor M2	CHRM2
kaempferol	Alpha-1B adrenergic receptor	ADRA1B
kaempferol	Gamma-aminobutyric acid receptor subunit alpha-1	GABRA1
kaempferol	DNA topoisomerase II	TOP2
kaempferol	Coagulation factor VII	F7
kaempferol	Calmodulin	CALM
kaempferol	Transcription factor p65	RELA
kaempferol	Inhibitor of nuclear factor kappa-B kinase subunit beta	IKBKB
kaempferol	RAC-alpha serine/threonine-protein kinase	AKT1
kaempferol	Apoptosis regulator Bcl-2	BCL2
kaempferol	Apoptosis regulator BAX	BAX
kaempferol	Tumor necrosis factor	TNF
kaempferol	Transcription factor AP-1	IL4
kaempferol	Activator of 90 kDa heat shock protein ATPase homolog 1	AHSA1

kaempferol	Caspase-3	CASP3
kaempferol	Mitogen-activated protein kinase 8	MAPK8
kaempferol	Xanthine dehydrogenase/oxidase	XDH
kaempferol	Interstitial collagenase	MMP1
kaempferol	Signal transducer and activator of transcription 1-alpha/beta	STAT1
kaempferol	Cell division control protein 2 homolog	CDK1
kaempferol	Peroxisome proliferator-activated receptor gamma	PPARG
kaempferol	Heme oxygenase 1	HMOX1
kaempferol	Cytochrome P450 3A4	CYP3A4
kaempferol	Cytochrome P450 1A2	CYP1A2
kaempferol	Cytochrome P450 1A1	CYP1A1
kaempferol	Intercellular adhesion molecule 1	ICAM1
kaempferol	E-selectin	SELE
kaempferol	Vascular cell adhesion protein 1	VCAM1
kaempferol	Nuclear receptor subfamily 1 group I member 2	NR2C2
kaempferol	Cytochrome P450 1B1	CYP1B1
kaempferol	Arachidonate 5-lipoxygenase	ALOX5
kaempferol	Hyaluronan synthase 2	HAS2
1,7-Dihydroxy-3,9-dimethoxy pterocarpene	Prostaglandin G/H synthase 2	PTGS2
1,7-Dihydroxy-3,9-dimethoxy pterocarpene	Retinoic acid receptor RXR-alpha	RXRA
1,7-Dihydroxy-3,9-dimethoxy pterocarpene	Heat shock protein HSP 90	HSP 90

1,7-Dihydroxy-3,9-dimethoxy pterocarpene	Trypsin-1	PRSS1
hederagenin	Progesterone receptor	PGR
hederagenin	Nuclear receptor coactivator 2	NCOA2
hederagenin	Muscarinic acetylcholine receptor M3	CHRNA7
hederagenin	Muscarinic acetylcholine receptor M1	CHRM1
hederagenin	Gamma-aminobutyric-acid receptor alpha-2 subunit	GABRA2
hederagenin	Gamma-aminobutyric-acid receptor alpha-3 subunit	GABRA3
hederagenin	Muscarinic acetylcholine receptor M2	CHRM2
hederagenin	Alpha-1B adrenergic receptor	ADRA1B
hederagenin	Gamma-aminobutyric acid receptor subunit alpha-1	GABRA1
hederagenin	Glutamate receptor 2	GRIA2
hederagenin	Gamma-aminobutyric-acid receptor subunit alpha-6	GABRA6
hederagenin	Gamma-aminobutyric-acid receptor alpha-5 subunit	GABRA5
hederagenin	Ig gamma-1 chain C region	IGHG1
hederagenin	Alcohol dehydrogenase 1B	ALDH1B1
hederagenin	Alcohol dehydrogenase 1C	ALDH3A1
hederagenin	Lysozyme	LYZ1
hederagenin	Nicotinate-nucleotide--dimethylb enzimidazole phosphoribosyltransferase	COBT

hederagenin	Prostaglandin G/H synthase 1	PTGS1
hederagenin	Sodium channel protein type 5 subunit alpha	SCN5A
hederagenin	Prostaglandin G/H synthase 2	PTGS2
hederagenin	Retinoic acid receptor RXR-alpha	RXRA
hederagenin	CGMP-inhibited 3',5'-cyclic phosphodiesterase A	PDE3A
hederagenin	Sodium-dependent noradrenaline transporter	SLC6A2
hederagenin	Cytochrome P450-cam	CYP21A2
9,10-dimethoxypterocarpan-3-O-β-D-glucoside	Prostaglandin G/H synthase 2	PTGS2
9,10-dimethoxypterocarpan-3-O-β-D-glucoside	DNA topoisomerase II	TOP2
9,10-dimethoxypterocarpan-3-O-β-D-glucoside	Nuclear receptor coactivator 2	NCOA2
(3S,8S,9S,10R,13R,14S,17R)-10,13-dimethyl-17-[(2R,5S)-5-propan-2-yloctan-2-yl]-2,3,4,7,8,9,11,12,14,15,16,17-dodecahydro-1H-cyclopenta[a]phenanthren-3-ol	Progesterone receptor	PGR
Bifendate	Prostaglandin G/H synthase 2	PTGS2
Bifendate	Vascular endothelial growth factor receptor 2	KDR
Bifendate	Hepatocyte growth factor receptor	MET
Bifendate	Heat shock protein HSP 90	HSP 90

Bifendate	Calcium-activated potassium channel subunit alpha 1	KCNMA1
Bifendate	Prostaglandin G/H synthase 1	PTGS1
Bifendate	DNA topoisomerase II	TOP2
beta-sitosterol	Progesterone receptor	PGR
beta-sitosterol	Nuclear receptor coactivator 2	NCOA2
beta-sitosterol	Prostaglandin G/H synthase 1	PTGS1
beta-sitosterol	Prostaglandin G/H synthase 2	PTGS2
beta-sitosterol	Heat shock protein HSP 90	HSP 90
beta-sitosterol	Phosphatidylinositol-4,5-bisphos phate 3-kinase catalytic subunit, gamma isoform	PIK3CG
beta-sitosterol	Potassium voltage-gated channel subfamily H member 2	KCNH2
beta-sitosterol	mRNA of PKA Catalytic Subunit C-alpha	PRKACA
beta-sitosterol	Dopamine D1 receptor	DRD1
beta-sitosterol	Muscarinic acetylcholine receptor M3	CHRNA7
beta-sitosterol	Muscarinic acetylcholine receptor M1	CHRM1
beta-sitosterol	Sodium channel protein type 5 subunit alpha	SCN5A
beta-sitosterol	Gamma-aminobutyric-acid receptor alpha-2 subunit	GABRA2
beta-sitosterol	Muscarinic acetylcholine receptor M4	CHRM4
beta-sitosterol	CGMP-inhibited 3',5'-cyclic phosphodiesterase A	PDE3A

beta-sitosterol	5-hydroxytryptamine 2A receptor	HTR2A
beta-sitosterol	Gamma-aminobutyric-acid receptor alpha-5 subunit	GABRA5
beta-sitosterol	Alpha-1A adrenergic receptor	ADRA1A
beta-sitosterol	Gamma-aminobutyric-acid receptor alpha-3 subunit	GABRA3
beta-sitosterol	Muscarinic acetylcholine receptor M2	CHRM2
beta-sitosterol	Alpha-1B adrenergic receptor	ADRA1B
beta-sitosterol	Beta-2 adrenergic receptor	ADRB2
beta-sitosterol	Neuronal acetylcholine receptor subunit alpha-2	CHRNA2
beta-sitosterol	Sodium-dependent serotonin transporter	SLC6A4
beta-sitosterol	Mu-type opioid receptor	OPRM1
beta-sitosterol	Gamma-aminobutyric acid receptor subunit alpha-1	GABRA1
beta-sitosterol	Neuronal acetylcholine receptor protein, alpha-7 chain	OPRM1
beta-sitosterol	Cytochrome P450-cam	CYP21A2
beta-sitosterol	Apoptosis regulator Bcl-2	BCL2
beta-sitosterol	Apoptosis regulator BAX	BAX
beta-sitosterol	Caspase-9	CASP9
beta-sitosterol	Transcription factor AP-1	IL4
beta-sitosterol	Caspase-3	CASP3
beta-sitosterol	Caspase-8	CASP8
beta-sitosterol	Protein kinase C alpha type	PRKCA
beta-sitosterol	Transforming growth factor beta-1	TGFB1

beta-sitosterol	Serum paraoxonase/arylesterase 1	PON1
beta-sitosterol	Microtubule-associated protein 2	MAP2
Stigmasterol	Progesterone receptor	PGR
Stigmasterol	Mineralocorticoid receptor	NR3C2
Stigmasterol	Nuclear receptor coactivator 2	NCOA2
Stigmasterol	Alcohol dehydrogenase 1C	ADH1C
Stigmasterol	Ig gamma-1 chain C region	IGHG1
Stigmasterol	Retinoic acid receptor RXR-alpha	RXRA
Stigmasterol	Nuclear receptor coactivator 1	NCOA1
Stigmasterol	Prostaglandin G/H synthase 1	PTGS1
Stigmasterol	Prostaglandin G/H synthase 2	PTGS2
Stigmasterol	Alpha-2A adrenergic receptor	ADRA2A
Stigmasterol	Sodium-dependent noradrenaline transporter	SLC6A2
Stigmasterol	Sodium-dependent dopamine transporter	SLC6A3
Stigmasterol	Beta-2 adrenergic receptor	ADRB2
Stigmasterol	Aldose reductase	AKR1B1
Stigmasterol	Urokinase-type plasminogen activator	PLAU
Stigmasterol	Leukotriene A-4 hydrolase	LTA4H
Stigmasterol	Amine oxidase [flavin-containing] B	PKIA
Stigmasterol	Amine oxidase [flavin-containing] A	MAOA
Stigmasterol	mRNA of PKA Catalytic Subunit C-alpha	PRKACA

Stigmasterol	Chymotrypsinogen B	CTRB1
Stigmasterol	Muscarinic acetylcholine receptor M3	CHRNA7
Stigmasterol	Muscarinic acetylcholine receptor M1	CHRM1
Stigmasterol	Beta-1 adrenergic receptor	ADRB1
Stigmasterol	Sodium channel protein type 5 subunit alpha	SCN5A
Stigmasterol	5-hydroxytryptamine 2A receptor	HTR2A
Stigmasterol	Alpha-1A adrenergic receptor	ADRA1A
Stigmasterol	Gamma-aminobutyric-acid receptor alpha-3 subunit	GABRA3
Stigmasterol	Muscarinic acetylcholine receptor M2	CHRM2
Stigmasterol	Alpha-1B adrenergic receptor	ADRA1B
Stigmasterol	Gamma-aminobutyric acid receptor subunit alpha-1	GABRA1
Stigmasterol	Neuronal acetylcholine receptor protein, alpha-7 chain	OPRM1

Supplementary Table S3 Proteins targeted by 17 active compounds of AR-RAS were enrichened in 95 signal pathways(P<0.001).

Gene Count % P-Value

Term	Gene Count	%	P-Value	Fold Enrichment	Benjamini
hsa05200:Pathways in cancer	53	27.31958763	1.30E-24	5.294789498	2.83E-22
hsa05161:Hepatitis B	34	17.5257732	3.97E-23	9.206112853	4.31E-21

hsa05212:Pancreatic cancer	22	11.34020619	1.97E-18	13.28846154	1.43E-16
hsa05223:Non-small cell lung cancer	19	9.793814433	6.70E-16	13.32081981	3.62E-14
hsa05219:Bladder cancer	17	8.762886598	8.26E-16	16.279102	3.38E-14
hsa05215:Prostate cancer	22	11.34020619	2.03E-15	9.815340909	7.23E-14
hsa04668:TNF signaling pathway	23	11.8556701	9.86E-15	8.518975129	3.06E-13
hsa05222:Small cell lung cancer	21	10.82474227	1.34E-14	9.69986631	3.64E-13
hsa05142:Chagas disease (American trypanosomiasis)	22	11.34020619	7.46E-14	8.305288462	1.80E-12
hsa05145:Toxoplasmosis	22	11.34020619	1.03E-12	7.319915254	2.23E-11
hsa05140:Leishmaniasis	18	9.278350515	1.05E-12	9.953585147	2.07E-11
hsa05210:Colorectal cancer	17	8.762886598	1.43E-12	10.76521261	2.59E-11
hsa04620:Toll-like receptor signaling pathway	20	10.30927835	1.16E-11	7.40780446	1.94E-10
hsa05220:Chronic myeloid leukemia	17	8.762886598	1.73E-11	9.270044192	2.68E-10
hsa05205:Proteoglycans in cancer	26	13.40206186	2.28E-11	5.103977273	3.29E-10
hsa04066:HIF-1 signaling pathway	19	9.793814433	2.71E-11	7.611897032	3.68E-10
hsa05164:Influenza A	24	12.37113	4.88E-1	5.4153605	6.23E-10

		402	1	02	
hsa05160:Hepatitis C	21	10.82474227	9.18E-11	6.199162679	1.11E-09
hsa05166:HTLV-I infection	28	14.43298969	1.81E-10	4.294211648	2.07E-09
hsa05213:Endometrial cancer	14	7.216494845	3.03E-10	10.57036713	3.29E-09
hsa04919:Thyroid hormone signaling pathway	19	9.793814433	3.72E-10	6.543560606	3.84E-09
hsa04151:PI3K-Akt signaling pathway	32	16.49484536	4.16E-10	3.641633729	4.10E-09
hsa05214:Glioma	15	7.731958763	5.26E-10	9.060314685	4.96E-09
hsa04660:T cell receptor signaling pathway	18	9.278350515	5.77E-10	6.861209179	5.22E-09
hsa04068:FoxO signaling pathway	20	10.30927835	7.87E-10	5.85990502	6.83E-09
hsa04115:p53 signaling pathway	15	7.731958763	8.07E-10	8.789857531	6.73E-09
hsa05169:Epstein-Barr virus infection	23	11.8556701	1.77E-09	4.752691388	1.42E-08
hsa04210:Apoptosis	14	7.216494845	3.18E-09	8.865469208	2.47E-08
hsa04380:Osteoclast differentiation	19	9.793814433	3.79E-09	5.694396253	2.84E-08
hsa05133:Pertussis	15	7.731958763	3.87E-09	7.852272727	2.80E-08
hsa05152:Tuberculosis	21	10.82474227	1.57E-08	4.658127889	1.10E-07

hsa04917:Prolactin signaling pathway	14	7.216494845	1.83E-08	7.741677337	1.24E-07
hsa05218:Melanoma	14	7.216494845	1.83E-08	7.741677337	1.24E-07
hsa04020:Calcium signaling pathway	21	10.82474227	1.91E-08	4.606081767	1.25E-07
hsa04010:MAPK signaling pathway	25	12.88659794	2.02E-08	3.849153298	1.29E-07
hsa04080:Neuroactive ligand-receptor interaction	26	13.40206186	2.32E-08	3.685182146	1.44E-07
hsa05144:Malaria	12	6.18556701	2.54E-08	9.615027829	1.53E-07
hsa04370:VEGF signaling pathway	13	6.701030928	2.83E-08	8.367175857	1.66E-07
hsa04012:ErbB signaling pathway	15	7.731958763	2.87E-08	6.769200627	1.64E-07
hsa04932:Non-alcoholic fatty liver disease (NAFLD)	19	9.793814433	3.73E-08	4.940171583	2.08E-07
hsa04071:Sphingolipid signaling pathway	17	8.762886598	4.52E-08	5.562026515	2.45E-07
hsa05146:Amoebiasis	16	8.24742268	5.41E-08	5.926243568	2.86E-07
hsa04931:Insulin resistance	16	8.24742268	6.99E-08	5.816498316	3.61E-07
hsa05143:African trypanosomiasis	10	5.154639175	7.98E-08	11.89738292	4.03E-07
hsa04621:NOD-like receptor signaling pathway	12	6.18556701	9.13E-08	8.566115702	4.50E-07
hsa05221:Acute myeloid	12	6.185567	1.11E-0	8.4131493	5.36E-07

leukemia		01	7	51	
hsa04510:Focal adhesion	21	10.82474 227	2.03E-0 7	4.0023720 21	9.58E-07
hsa04064:NF-kappa B signaling pathway	14	7.216494 845	2.26E-0 7	6.3179205 85	1.04E-06
hsa05230:Central carbon metabolism in cancer	12	6.185567 01	4.66E-0 7	7.3615056 82	2.10E-06
hsa05321:Inflammatory bowel disease (IBD)	12	6.185567 01	4.66E-0 7	7.3615056 82	2.10E-06
hsa04725:Cholinergic synapse	15	7.731958 763	6.56E-0 7	5.3055896 81	2.91E-06
hsa05206:MicroRNAs in cancer	24	12.37113 402	6.77E-0 7	3.3062200 96	2.94E-06
hsa05134:Legionellosis	11	5.670103 093	7.66E-0 7	7.9976851 85	3.26E-06
hsa04014:Ras signaling pathway	21	10.82474 227	9.10E-0 7	3.6481798 07	3.80E-06
hsa05132:Salmonella infection	13	6.701030 928	9.68E-0 7	6.1493702 08	3.96E-06
hsa05162:Measles	16	8.247422 68	1.12E-0 6	4.7231715 65	4.48E-06
hsa05020:Prion diseases	9	4.639175 258	1.17E-0 6	10.707644 63	4.60E-06
hsa05323:Rheumatoid arthritis	13	6.701030 928	1.84E-0 6	5.7999741 74	7.12E-06
hsa04024:cAMP signaling pathway	19	9.793814 433	2.25E-0 6	3.7675045 91	8.57E-06
hsa04110:Cell cycle	15	7.731958 763	2.55E-0 6	4.7493585 04	9.54E-06

hsa05014:Amyotrophic lateral sclerosis (ALS)	10	5.154639175	3.58E-06	7.852272727	1.32E-05
hsa05202:Transcriptional misregulation in cancer	17	8.762886598	4.65E-06	3.972876082	1.68E-05
hsa05120:Epithelial cell signaling in Helicobacter pylori infection	11	5.670103093	5.97E-06	6.445895522	2.12E-05
hsa04915:Estrogen signaling pathway	13	6.701030928	6.49E-06	5.155532599	2.27E-05
hsa04662:B cell receptor signaling pathway	11	5.670103093	7.84E-06	6.259057971	2.70E-05
hsa04723:Retrograde endocannabinoid signaling	13	6.701030928	8.01E-06	5.053442844	2.72E-05
hsa04920:Adipocytokine signaling pathway	11	5.670103093	8.94E-06	6.169642857	2.99E-05
hsa04722:Neurotrophin signaling pathway	14	7.216494845	9.34E-06	4.580492424	3.07E-05
hsa04914:Progesterone-mediated oocyte maturation	12	6.18556701	1.06E-05	5.415360502	3.43E-05
hsa04540:Gap junction	12	6.18556701	1.18E-05	5.353822314	3.77E-05
hsa05168:Herpes simplex infection	17	8.762886598	1.40E-05	3.647230502	4.39E-05
hsa04726:Serotonergic synapse	13	6.701030928	2.13E-05	4.598177723	6.59E-05
hsa05031:Amphetamine addiction	10	5.154639175	3.73E-05	5.94869146	1.14E-04
hsa04910:Insulin signaling pathway	14	7.216494845	4.23E-05	3.983036891	1.27E-04

hsa05231:Choline metabolism in cancer	12	6.18556701	4.42E-05	4.664716472	1.31E-04
hsa04664:Fc epsilon RI signaling pathway	10	5.154639175	4.75E-05	5.773729947	1.39E-04
hsa05203:Viral carcinogenesis	17	8.762886598	5.69E-05	3.255820399	1.65E-04
hsa04622:RIG-I-like receptor signaling pathway	10	5.154639175	6.01E-05	5.608766234	1.72E-04
hsa04060:Cytokine-cytokine receptor interaction	18	9.278350515	6.59E-05	3.072628458	1.86E-04
hsa04062:Chemokine signaling pathway	16	8.24742268	6.76E-05	3.377321603	1.88E-04
hsa05216:Thyroid cancer	7	3.608247423	7.09E-05	9.476880878	1.95E-04
hsa04923:Regulation of lipolysis in adipocytes	9	4.639175258	7.26E-05	6.309862013	1.97E-04
hsa04022:cGMP-PKG signaling pathway	15	7.731958763	7.30E-05	3.547713582	1.96E-04
hsa05032:Morphine addiction	11	5.670103093	9.16E-05	4.745879121	2.42E-04
hsa04912:GnRH signaling pathway	11	5.670103093	9.16E-05	4.745879121	2.42E-04
hsa04930:Type II diabetes mellitus	8	4.12371134	1.82E-04	6.543560606	4.75E-04
hsa04913:Ovarian steroidogenesis	8	4.12371134	2.07E-04	6.410018553	5.36E-04
hsa05211:Renal cell carcinoma	9	4.639175258	2.12E-04	5.436188811	5.42E-04
hsa04970:Salivary secretion	10	5.154639	3.00E-0	4.5652748	7.57E-04

		175	4	41	
hsa04261:Adrenergic signaling in cardiomyocytes	13	6.701030928	3.08E-04	3.495874844	7.67E-04
hsa04728:Dopaminergic synapse	12	6.18556701	3.74E-04	3.680752841	9.22E-04
hsa05033:Nicotine addiction	7	3.608247423	4.54E-04	6.870738636	0.001107733
hsa04150:mTOR signaling pathway	8	4.12371134	5.99E-04	5.415360502	0.001444647
hsa04921:Oxytocin signaling pathway	13	6.701030928	6.32E-04	3.230365362	0.001506912
hsa05204:Chemical carcinogenesis	9	4.639175258	8.84E-04	4.416903409	0.002083168

Supplementary Table S4 TBI specific proteins

Protein name	Gene name	Protein name	Gene name
Nitric oxide synthase, inducible	NOS2	*Ornithine decarboxylase*	ODC1
Prostaglandin G/H synthase 1	PTGS1	*Xanthine dehydrogenase/oxidase*	XDH
Dopamine D1 receptor	DRD1	*Caspase-8*	CASP8
Muscarinic acetylcholine receptor M3	CHRM3	*DNA topoisomerase 1*	TOP1
Thrombin	F2	*RAF proto-oncogene serine/threonine-protein kinase*	RAF1
Potassium voltage-gated channel subfamily H	KCNH2	*Superoxide dismutase [Cu-Zn]*	SOD1

member 2			
Muscarinic acetylcholine receptor M1	CHRM1	*Protein kinase C alpha type*	PRKCA
Estrogen receptor	ESR1	*Interstitial collagenase*	MMP1
Androgen receptor	AR	*Hypoxia-inducible factor 1-alpha*	HIF1A
Beta-1 adrenergic receptor	ADRB1	*Signal transducer and activator of transcription 1-alpha/beta*	STAT1
Sodium channel protein type 5 subunit alpha	SCN5A	*Protein CBFA2T1*	RUNX1T1
Peroxisome proliferator activated receptor gamma	PPARG	*Probable E3 ubiquitin-protein ligase HERC5*	HERC5
Coagulation factor Xa	F10	*Cell division control protein 2 homolog*	CDK1
Muscarinic acetylcholine receptor M5	CHRM5	*78 kDa glucose-regulated protein*	HSPA5
Prostaglandin G/H synthase 2	PTGS2	*Receptor tyrosine-protein kinase erbB-2*	ERBB2
Nitric-oxide synthase, endothelial	NOS3	*Peroxisome proliferator-activated receptor gamma*	PPARG
Alpha-2C adrenergic receptor	ADRA2C	*Acetyl-CoA carboxylase 1*	ACACA
Muscarinic acetylcholine receptor M4	CHRM4	*Heme oxygenase 1*	HMOX1
Retinoic acid receptor RXR-alpha	RXRA	*Cytochrome P450 3A4*	CYP3A4

Delta-type opioid receptor	OPRD1	*Cytochrome P450 1A2*	CYP1A2
CGMP-inhibited 3',5'-cyclic phosphodiesterase A	PDE3A	*Caveolin-1*	CAV1
5-hydroxytryptamine 2A receptor	HTR2A	*Myc proto-oncogene protein*	MYC
Alpha-1A adrenergic receptor	ADRA1A	*Tissue factor*	F3
Muscarinic acetylcholine receptor M2	CHRM2	*Gap junction alpha-1 protein*	GJA1
Alpha-1B adrenergic receptor	ADRA1B	*Cytochrome P450 1A1*	CYP1A1
Sodium-dependent dopamine transporter	SLC6A3	*Intercellular adhesion molecule 1*	ICAM1
Beta-2 adrenergic receptor	ADRB2	*Interleukin-1 beta*	IL1B
Alpha-1D adrenergic receptor	ADRA1D	*C-C motif chemokine 2*	CCL2
Sodium-dependent serotonin transporter	SLC6A4	*E-selectin*	SELE
Estrogen receptor beta	ESRRB	*Vascular cell adhesion protein 1*	VCAM1
Gamma-aminobutyric acid receptor subunit alpha-1	GABRA1	*Prostaglandin E2 receptor EP3 subtype*	PTGER3
Dipeptidyl peptidase IV	DPP4	*Interleukin-8*	CXCL8
Mitogen-activated protein kinase 14	MAPK14	*Protein kinase C beta type*	PRKCB
Glycogen synthase	GSK3B	*Baculoviral IAP*	BIRC5

kinase-3 beta		*repeat-containing protein 5*	
Heat shock protein HSP 90	HSP 90	*Dual oxidase 2*	DUOX2
Cell division protein kinase 2	CDK2	*Nitric oxide synthase, endothelial*	NOS3
Serine/threonine-protein kinase Chk1	CHEK1	*Heat shock protein beta-1*	HSPB1
mRNA of PKA Catalytic Subunit C-alpha	PRKACA	*Transforming growth factor beta-1*	TGFB1
Retinoic acid receptor RXR-beta	RXRB	*Estrogen sulfotransferase*	SULT1E1
Trypsin-1	PRSS1	*Maltase-glucoamylase, intestinal*	MGAM
Proto-oncogene serine/threonine-protein kinase Pim-1	PIM1	*Interleukin-2*	IL2
Cyclin-A2	CCNA2	*Nuclear receptor subfamily 1 group I member 2*	NR2C2
Nuclear receptor coactivator 2	NCOA2	*Cytochrome P450 1B1*	CYP1B1
Calcium-activated potassium channel subunit alpha 1	KCNMA1	*G2/mitotic-specific cyclin-B1*	CCNB1
Calmodulin	CALM	*Tissue-type plasminogen activator*	PLAT
Nitric oxide synthase, inducible	NOS2	*Thrombomodulin*	THBD
Prostaglandin G/H synthase 1	PTGS1	*Plasminogen activator inhibitor 1*	SERPINE 1

Muscarinic acetylcholine receptor M1	CHRM1	*Collagen alpha-1(I) chain*	COL1A1
Estrogen receptor	ESR2	*Interferon gamma*	IFNG
Androgen receptor	AR	*Arachidonate 5-lipoxygenase*	ALOX5
Peroxisome proliferator activated receptor gamma	PPARG	*Phosphatidylinositol-3,4,5-trisphosphate 3-phosphatase and dual-specificity protein phosphatase PTEN*	PTEN
Prostaglandin G/H synthase 2	PTGS2	*Interleukin-1 alpha*	IL1A
Retinoic acid receptor RXR-alpha	RXRA	*Myeloperoxidase*	MPO
CGMP-inhibited 3',5'-cyclic phosphodiesterase A	PDE3A	*DNA topoisomerase 2-alpha*	TOP2A
Alpha-1A adrenergic receptor	ADRA1A	*Neutrophil cytosol factor 1*	NCF1
Sodium-dependent dopamine transporter	SLC6A3	*ATP-binding cassette sub-family G member 2*	ABCG2
Beta-2 adrenergic receptor	ADRB2	*Hyaluronan synthase 2*	HAS2
Sodium-dependent serotonin transporter	SLC6A4	*Glutathione S-transferase P*	GSTP1
Estrogen receptor beta	MAOB	*Nuclear factor erythroid 2-related factor 2*	NFE2L2
Dipeptidyl peptidase IV	DPP4	*NAD(P)H dehydrogenase [quinone] 1*	NQO1
Mitogen-activated protein	MAPK14	*Poly [ADP-ribose]*	PARP1

kinase 14		*polymerase 1*	
Glycogen synthase kinase-3 beta	GSK3B	*Aryl hydrocarbon receptor*	AHR
Heat shock protein HSP 90	HSP90	*26S proteasome non-ATPase regulatory subunit 3*	PSMD3
Cell division protein kinase 2	CDK2	*Solute carrier family 2, facilitated glucose transporter member 4*	SLC2A4
Amine oxidase [flavin-containing] B	PKIA	*Collagen alpha-1(III) chain*	COL3A1
Serine/threonine-protein kinase Chk1	CHEK1	*DNA gyrase subunit B*	GYRB
mRNA of PKA Catalytic Subunit C-alpha	PRKACA	*C-X-C motif chemokine 11*	CXCL11
Trypsin-1	PRSS1	*C-X-C motif chemokine 2*	CXCL2
Proto-oncogene serine/threonine-protein kinase Pim-1	PIM1	*DDB1- and CUL4-associated factor 5*	DCAF5
Cyclin-A2	CCNA2	*Nuclear receptor subfamily 1 group I member 3*	NR1I3
Calmodulin	CALM	*Serine/threonine-protein kinase Chk2*	CHEK2
cAMP-dependent protein kinase inhibitor alpha	ACHE	*Insulin receptor*	INSR
Thrombin	F2	*Claudin-4*	CLDN4
Nitric-oxide synthase, endothelial	NOS3	*Peroxisome proliferator-activated receptor alpha*	PPARA

Acetylcholinesterase	LACTB2	*Peroxisome proliferator-activated receptor delta*	PPARD
Beta-lactamase	JUN	*Heat shock factor protein 1*	HSF1
Transcription factor AP-1	IL4	*C-reactive protein*	CRP
Peroxisome proliferator-activated receptor gamma	PPARG	*C-X-C motif chemokine 10*	CXCL10
Interleukin-4	SIRT1	*Inhibitor of nuclear factor kappa-B kinase subunit alpha*	CHUK
NAD-dependent deacetylase sirtuin-1	ATP5F1B	*Osteopontin*	SPP1
ATP synthase subunit beta, mitochondrial	MT-ND6	*Runt-related transcription factor 2*	RUNX2
NADH-ubiquinone oxidoreductase chain 6	HSD3B2	*Ras association domain-containing protein 1*	RASSF1
3 beta-hydroxysteroid dehydrogenase/Delta 5-->4-isomerase type 2	HSD3B1	*Transcription factor E2F1*	E2F1
3 beta-hydroxysteroid dehydrogenase/Delta 5-->4-isomerase type 1	HTR3A	*Transcription factor E2F2*	E2F2
Cell division protein kinase 2	CDK2	*Prostatic acid phosphatase*	ACPP
Thrombin	F2	*Cathepsin D*	CTSD
Glycogen synthase kinase-3 beta	GSK3B	*Insulin-like growth factor-binding protein 3*	IGFBP3

Nitric oxide synthase, inducible	NOS2	*Insulin-like growth factor II*	IGF2
Prostaglandin G/H synthase 1	PTGS1	*CD40 ligand*	CD40LG
Muscarinic acetylcholine receptor M3	CHRNA7	*Interferon regulatory factor 1*	IRF1
Thrombin	F2	*Receptor tyrosine-protein kinase erbB-3*	ERBB3
Muscarinic acetylcholine receptor M1	CHRM1	*Serum paraoxonase/arylesterase 1*	PON1
Estrogen receptor	ESR1	*Type I iodothyronine deiodinase*	DIO1
Sodium channel protein type 5 subunit alpha	SCN5A	*Procollagen C-endopeptidase enhancer 1*	PCOLCE
Prostaglandin G/H synthase 2	PTGS2	*Puromycin-sensitive aminopeptidase*	NPEPPS
5-hydroxytryptamine receptor 3A	NCOA1	*Hexokinase-2*	HK2
Retinoic acid receptor RXR-alpha	PGR	*Homeobox protein Nkx-3.1*	NKX3-1
Acetylcholinesterase	LACTB2	*Ras GTPase-activating protein 1*	RASA1
Alpha-1B adrenergic receptor	ADRA1B	*Peroxidase C1A*	PRXC1A
Beta-2 adrenergic receptor	ADRB2	*Glutathione S-transferase Mu 1*	GSTM1
Alpha-1D adrenergic receptor	ADRA1D	*Glutathione S-transferase Mu 2*	GSTM2

Gamma-aminobutyric acid receptor subunit alpha-1	GABRA1	*Nitric oxide synthase, inducible*	NOS2
Heat shock protein HSP 90	HSP 90	*Prostaglandin G/H synthase 1*	PTGS1
Neuronal acetylcholine receptor protein, alpha-7 chain	OPRM1	*Androgen receptor*	AR
Trypsin-1	PRSS1	*Peroxisome proliferator activated receptor gamma*	PPARG
Nuclear receptor coactivator 2	NCOA2	*Prostaglandin G/H synthase 2*	PTGS2
Nuclear receptor coactivator 1	NCOA1	*Heat shock protein HSP 90*	HSP 90
Calmodulin	CALM	*Phosphatidylinositol-4,5-bis phosphate 3-kinase catalytic subunit, gamma isoform*	PIK3CG
Muscarinic acetylcholine receptor M4	CHRM4	*mRNA of PKA Catalytic Subunit C-alpha*	PRKACA
Progesterone receptor	PGR	*Nuclear receptor coactivator 2*	NCOA2
Nitric oxide synthase, inducible	NOS2	*Dipeptidyl peptidase IV*	DPP4
Prostaglandin G/H synthase 1	PTGS1	*Trypsin-1*	PRSS1
Muscarinic acetylcholine receptor M3	CHRNA7	*Progesterone receptor*	PGR
Thrombin	F2	*Thrombin*	F2

Muscarinic acetylcholine receptor M1	CHRM1	*Muscarinic acetylcholine receptor M1*	CHRM1
Estrogen receptor	ESR1	*Nitric-oxide synthase, endothelial*	NOS3
Beta-1 adrenergic receptor	ADRB1	*Gamma-aminobutyric-acid receptor alpha-2 subunit*	GABRA2
Sodium channel protein type 5 subunit alpha	SCN5A	*Acetylcholinesterase*	LACTB2
Prostaglandin G/H synthase 2	PTGS2	*Sodium-dependent noradrenaline transporter*	SLC6A2
Nitric-oxide synthase, endothelial	NOS3	*Muscarinic acetylcholine receptor M2*	CHRM2
5-hydroxytryptamine receptor 3A	HTR3A	*Alpha-1B adrenergic receptor*	ADRA1B
Alpha-2C adrenergic receptor	ADRA2C	*Gamma-aminobutyric acid receptor subunit alpha-1*	GABRA1
Retinoic acid receptor RXR-alpha	RXRA	*DNA topoisomerase II*	TOP2
Acetylcholinesterase	LACTB2	*Coagulation factor VII*	F7
CGMP-inhibited 3',5'-cyclic phosphodiesterase A	PDE3A	*Calmodulin*	CALM
Alpha-1B adrenergic receptor	ADRA1B	*Transcription factor p65*	RELA
Beta-2 adrenergic receptor	ADRB2	*Inhibitor of nuclear factor kappa-B kinase subunit beta*	IKBKB
Alpha-1D adrenergic receptor	ADRA1D	*RAC-alpha serine/threonine-protein kinase*	AKT1

Mu-type opioid receptor	OPRM1	*Apoptosis regulator Bcl-2*	BCL2
Gamma-aminobutyric acid receptor subunit alpha-1	GABRA1	*Apoptosis regulator BAX*	BAX
Trypsin-1	PRSS1	*Tumor necrosis factor*	TNF
Nuclear receptor coactivator 2	NCOA2	*Transcription factor AP-1*	IL4
Calmodulin	CALM	*Activator of 90 kDa heat shock protein ATPase homolog 1*	AHSA1
Nitric oxide synthase, inducible	NOS2	*Caspase-3*	CASP3
Prostaglandin G/H synthase 1	PTGS1	*Mitogen-activated protein kinase 8*	MAPK8
Androgen receptor	AR	*Xanthine dehydrogenase/oxidase*	XDH
Sodium channel protein type 5 subunit alpha	SCN5A	*Interstitial collagenase*	MMP1
Prostaglandin G/H synthase 2	PTGS2	*Signal transducer and activator of transcription 1-alpha/beta*	STAT1
Estrogen receptor beta	MAOB	*Cell division control protein 2 homolog*	CDK1
Dipeptidyl peptidase IV	DPP4	*Peroxisome proliferator-activated receptor gamma*	PPARG
Heat shock protein HSP 90	HSP 90	*Heme oxygenase 1*	HMOX1
Cell division protein	CDK2	*Cytochrome P450 3A4*	CYP3A4

kinase 2			
Serine/threonine-protein kinase Chk1	CHEK1	*Cytochrome P450 1A2*	CYP1A2
Trypsin-1	PRSS1	*Cytochrome P450 1A1*	CYP1A1
Nuclear receptor coactivator 2	NCOA2	*Intercellular adhesion molecule 1*	ICAM1
Calmodulin	CALM	*E-selectin*	SELE
Nitric oxide synthase, inducible	NOS2	*Vascular cell adhesion protein 1*	VCAM1
Prostaglandin G/H synthase 1	PTGS1	*Nuclear receptor subfamily 1 group I member 2*	NR2C2
Estrogen receptor	ESR1	*Cytochrome P450 1B1*	CYP1B1
Androgen receptor	AR	*Arachidonate 5-lipoxygenase*	ALOX5
Peroxisome proliferator activated receptor gamma	PPARG	*Hyaluronan synthase 2*	HAS2
Prostaglandin G/H synthase 2	PTGS2	*Prostaglandin G/H synthase 2*	PTGS2
mRNA of Protein-tyrosine phosphatase, non-receptor type 1	PTPN1	*Retinoic acid receptor RXR-alpha*	RXRA
Estrogen receptor beta	MAOB	*Heat shock protein HSP 90*	HSP 90
Dipeptidyl peptidase IV	DPP4	*Trypsin-1*	PRSS1
Mitogen-activated protein kinase 14	MAPK14	*Progesterone receptor*	PGR
Glycogen synthase kinase-3 beta	GSK3B	*Nuclear receptor coactivator 2*	NCOA2
Heat shock protein HSP 90	HSP 90	*Muscarinic acetylcholine receptor M3*	CHRNA7

Cell division protein kinase 2	CDK2	*Muscarinic acetylcholine receptor M1*	CHRM1
Phosphatidylinositol-4,5-bisphosphate 3-kinase catalytic subunit, gamma isoform	PIK3CG	*Gamma-aminobutyric-acid receptor alpha-2 subunit*	GABRA2
mRNA of PKA Catalytic Subunit C-alpha	PRKACA	*Gamma-aminobutyric-acid receptor alpha-3 subunit*	GABRA3
Trypsin-1	PRSS1	*Muscarinic acetylcholine receptor M2*	CHRM2
Proto-oncogene serine/threonine-protein kinase Pim-1	PIM1	*Alpha-1B adrenergic receptor*	ADRA1B
Cyclin-A2	CCNA2	*Gamma-aminobutyric acid receptor subunit alpha-1*	GABRA1
Nuclear receptor coactivator 2	NCOA2	*Glutamate receptor 2*	GRIA2
Calmodulin	CALM	*Gamma-aminobutyric-acid receptor subunit alpha-6*	GABRA6
Glycogen phosphorylase, muscle form	PYGM	*Gamma-aminobutyric-acid receptor alpha-5 subunit*	GABRA5
Peroxisome proliferator activated receptor delta	PPARD	*Ig gamma-1 chain C region*	IGHG1
Serine/threonine-protein kinase Chk1	CHEK1	*Alcohol dehydrogenase 1B*	ALDH1B1
Aldose reductase	AKR1B1	*Alcohol dehydrogenase 1C*	ALDH3A1
Nuclear receptor coactivator 1	NCOA1	*Lysozyme*	LYZ1
Coagulation factor VII	F7	*Nicotinate-nucleotide--dime*	COBT

		thylbenzimidazole phosphoribosyltransferase	
Thrombin	F2	*Prostaglandin G/H synthase 1*	PTGS1
Nitric-oxide synthase, endothelial	NOS3	*Sodium channel protein type 5 subunit alpha*	SCN5A
Acetylcholinesterase	LACTB2	*Prostaglandin G/H synthase 2*	PTGS2
Gamma-aminobutyric acid receptor subunit alpha-1	GABRA1	*Retinoic acid receptor RXR-alpha*	RXRA
Amine oxidase [flavin-containing] B	PKIA	*CGMP-inhibited 3',5'-cyclic phosphodiesterase A*	PDE3A
Glutamate receptor 2	GRIA2	*Sodium-dependent noradrenaline transporter*	SLC6A2
Cytochrome P450-cam	CAMC	*Cytochrome P450-cam*	CYP21A2
Transcription factor p65	RELA	*Prostaglandin G/H synthase 2*	PTGS2
Xanthine dehydrogenase/oxidase	XDH	*DNA topoisomerase II*	TOP2
Neutrophil cytosol factor 1	NCOA1	*Nuclear receptor coactivator 2*	NCOA2
Oxidized low-density lipoprotein receptor 1	OLR1	*Progesterone receptor*	PGR
DNA topoisomerase II	TOP2	*Prostaglandin G/H synthase 2*	PTGS2
Nitric oxide synthase, inducible	NOS2	*Vascular endothelial growth factor receptor 2*	KDR
Prostaglandin G/H	PTGS1	*Hepatocyte growth factor*	MET

synthase 1		*receptor*	
Estrogen receptor	ESR1	*Heat shock protein HSP 90*	HSP 90
Androgen receptor	AR	*Calcium-activated potassium channel subunit alpha 1*	KCNMA1
Peroxisome proliferator activated receptor gamma	PPARG	*Prostaglandin G/H synthase 1*	PTGS1
Prostaglandin G/H synthase 2	PTGS2	*DNA topoisomerase II*	TOP2
Retinoic acid receptor RXR-alpha	RXRA	*Progesterone receptor*	PGR
CGMP-inhibited 3',5'-cyclic phosphodiesterase A	PDE3A	*Nuclear receptor coactivator 2*	NCOA2
Estrogen receptor beta	MAOB	*Prostaglandin G/H synthase 1*	PTGS1
Dipeptidyl peptidase IV	DPP4	*Prostaglandin G/H synthase 2*	PTGS2
Mitogen-activated protein kinase 14	MAPK14	*Heat shock protein HSP 90*	HSP 90
Glycogen synthase kinase-3 beta	GSK3B	*Phosphatidylinositol-4,5-bisphosphate 3-kinase catalytic subunit, gamma isoform*	PIK3CG
Heat shock protein HSP 90	HSP 90	*Potassium voltage-gated channel subfamily H member 2*	KCNH2
Cell division protein kinase 2	CDK2	*mRNA of PKA Catalytic Subunit C-alpha*	PRKACA

Serine/threonine-protein kinase Chk1	CHEK1	*Dopamine D1 receptor*	DRD1
mRNA of PKA Catalytic Subunit C-alpha	PRKACA	*Muscarinic acetylcholine receptor M3*	CHRNA7
Trypsin-1	PRSS1	*Muscarinic acetylcholine receptor M1*	CHRM1
Proto-oncogene serine/threonine-protein kinase Pim-1	PIM1	*Sodium channel protein type 5 subunit alpha*	SCN5A
Cyclin-A2	CCNA2	*Gamma-aminobutyric-acid receptor alpha-2 subunit*	GABRA2
Nuclear receptor coactivator 2	NCOA2	*Muscarinic acetylcholine receptor M4*	CHRM4
Calmodulin	CALM	*CGMP-inhibited 3',5'-cyclic phosphodiesterase A*	PDE3A
Beta-2 adrenergic receptor	ADRB2	*5-hydroxytryptamine 2A receptor*	HTR2A
Prostaglandin G/H synthase 1	PTGS1	*Gamma-aminobutyric-acid receptor alpha-5 subunit*	GABRA5
Androgen receptor	AR	*Alpha-1A adrenergic receptor*	ADRA1A
Peroxisome proliferator activated receptor gamma	PPARG	*Gamma-aminobutyric-acid receptor alpha-3 subunit*	GABRA3
Prostaglandin G/H synthase 2	PTGS2	*Muscarinic acetylcholine receptor M2*	CHRM2
Heat shock protein HSP 90	HSP 90	*Alpha-1B adrenergic receptor*	ADRA1B
Phosphatidylinositol-4,5-bisphosphate 3-kinase	PIK3CG	*Beta-2 adrenergic receptor*	ADRB2

catalytic subunit, gamma isoform			
Nuclear receptor coactivator 2	NCOA2	*Neuronal acetylcholine receptor subunit alpha-2*	CHRNA2
Dipeptidyl peptidase IV	DPP4	*Sodium-dependent serotonin transporter*	SLC6A4
Aldose reductase	AKR1B1	*Mu-type opioid receptor*	OPRM1
Trypsin-1	PRSS1	*Gamma-aminobutyric acid receptor subunit alpha-1*	GABRA1
DNA topoisomerase II	TOP2	*Neuronal acetylcholine receptor protein, alpha-7 chain*	OPRM1
Thrombin	F2	*Cytochrome P450-cam*	CYP21A2
Potassium voltage-gated channel subfamily H member 2	KCNH2	*Apoptosis regulator Bcl-2*	BCL2
Sodium channel protein type 5 subunit alpha	SCN5A	*Apoptosis regulator BAX*	BAX
Coagulation factor Xa	F10	*Caspase-9*	CASP9
Beta-2 adrenergic receptor	ADRB2	*Transcription factor AP-1*	IL4
Stromelysin-1	MMP3	*Caspase-3*	CASP3
mRNA of PKA Catalytic Subunit C-alpha	PRKACA	*Caspase-8*	CASP8
Coagulation factor VII	F7	*Protein kinase C alpha type*	PRKCA
Nitric-oxide synthase, endothelial	NOS3	*Transforming growth factor beta-1*	TGFB1
Retinoic acid receptor RXR-alpha	RXRA	*Serum paraoxonase/arylesterase 1*	PON1

Acetylcholinesterase	LACTB2	*Microtubule-associated protein 2*	MAP2
Gamma-aminobutyric acid receptor subunit alpha-1	GABRA1	*Progesterone receptor*	PGR
Amine oxidase [flavin-containing] B	PKIA	*Mineralocorticoid receptor*	NR3C2
Transcription factor p65	RELA	*Nuclear receptor coactivator 2*	NCOA2
Epidermal growth factor receptor	EGFR	*Alcohol dehydrogenase 1C*	ADH1C
RAC-alpha serine/threonine-protein kinase	AKT1	*Ig gamma-1 chain C region*	IGHG1
Vascular endothelial growth factor A	VEGFA	*Retinoic acid receptor RXR-alpha*	RXRA
G1/S-specific cyclin-D1	CCND1	*Nuclear receptor coactivator 1*	NCOA1
Apoptosis regulator Bcl-2	BCL2	*Prostaglandin G/H synthase 1*	PTGS1
Bcl-2-like protein 1	BCL2L1	*Prostaglandin G/H synthase 2*	PTGS2
Proto-oncogene c-Fos	FOS	*Alpha-2A adrenergic receptor*	ADRA2A
Cyclin-dependent kinase inhibitor 1	CDKN1	*Sodium-dependent noradrenaline transporter*	SLC6A2
Eukaryotic translation initiation factor 6	EIF6	*Sodium-dependent dopamine transporter*	SLC6A3
Apoptosis regulator BAX	BAX	*Beta-2 adrenergic receptor*	ADRB2

Caspase-9	CASP9	*Aldose reductase*	AKR1B1
Urokinase-type plasminogen activator	PLAU	*Urokinase-type plasminogen activator*	PLAU
72 kDa type IV collagenase	MMP2	*Leukotriene A-4 hydrolase*	LTA4H
Matrix metalloproteinase-9	MMP9	*Amine oxidase [flavin-containing] B*	PKIA
Mitogen-activated protein kinase 1	MAPK1	*Amine oxidase [flavin-containing] A*	MAOA
Interleukin-10	IL10	*mRNA of PKA Catalytic Subunit C-alpha*	PRKACA
Pro-epidermal growth factor	EGF	*Chymotrypsinogen B*	CTRB1
Retinoblastoma-associate d protein	RB1	*Muscarinic acetylcholine receptor M3*	CHRNA7
Tumor necrosis factor	TNF	*Muscarinic acetylcholine receptor M1*	CHRM1
Transcription factor AP-1	IL4	*Beta-1 adrenergic receptor*	ADRB1
Interleukin-6	IL6	*Sodium channel protein type 5 subunit alpha*	SCN5A
Cyclin-dependent kinase inhibitor 2A, isoforms 1/2/3	CDKN2A	*5-hydroxytryptamine 2A receptor*	HTR2A
Activator of 90 kDa heat shock protein ATPase homolog 1	AHSA1	*Alpha-1A adrenergic receptor*	ADRA1A
Caspase-3	CASP3	*Gamma-aminobutyric-acid receptor alpha-3 subunit*	GABRA3
Cellular tumor antigen	TP53	*Muscarinic acetylcholine*	CHRM2

p53		*receptor M2*	
ETS domain-containing protein Elk-1	ELK1	*Alpha-1B adrenergic receptor*	ADRA1B
NF-kappa-B inhibitor alpha	NFKBIA	*Gamma-aminobutyric acid receptor subunit alpha-1*	GABRA1
NADPH--cytochrome P450 reductase	POR	*Neuronal acetylcholine receptor protein, alpha-7 chain*	OPRM1

Supplementary Table S5 89 signal pathways related to AR-RAS for treating TBI

Term	Count	%	P-Value	Fold Enrichment	Benjamini
hsa05161:Hepatitis B	11	34.375	1.73E-10	17.47356322	2.82E-08
hsa05145:Toxoplasmosis	10	31.25	6.83E-10	19.51977401	5.57E-08
hsa05200:Pathways in cancer	13	40.625	2.08E-08	7.619168787	1.13E-06
hsa05152:Tuberculosis	10	31.25	2.50E-08	13.01318267	1.02E-06
hsa04066:HIF-1 signaling pathway	8	25	1.12E-07	18.80272109	3.67E-06
hsa05164:Influenza A	9	28.125	3.76E-07	11.9137931	1.02E-05
hsa04071:Sphingolipid	8	25	4.54E-07	15.355555	1.06E-05

signaling pathway				56	
hsa04010:MAPK signaling pathway	10	31.25	5.78E-07	9.0326797 39	1.18E-05
hsa05223:Non-small cell lung cancer	6	18.75	2.98E-06	24.678571 43	5.40E-05
hsa04150:mTOR signaling pathway	6	18.75	3.56E-06	23.827586 21	5.80E-05
hsa05142:Chagas disease (American trypanosomiasis)	7	21.87 5	3.61E-06	15.503205 13	5.34E-05
hsa04210:Apoptosis	6	18.75	4.97E-06	22.290322 58	6.75E-05
hsa05210:Colorectal cancer	6	18.75	4.97E-06	22.290322 58	6.75E-05
hsa04725:Cholinergic synapse	7	21.87 5	5.27E-06	14.525525 53	6.61E-05
hsa05212:Pancreatic cancer	6	18.75	6.29E-06	21.261538 46	7.32E-05
hsa04380:Osteoclast differentiation	7	21.87 5	1.37E-05	12.307888 04	1.49E-04
hsa05205:Proteoglycans in cancer	8	25	1.39E-05	9.2133333 33	1.42E-04
hsa05160:Hepatitis C	7	21.87 5	1.50E-05	12.122807 02	1.44E-04
hsa04012:ErbB signaling pathway	6	18.75	2.64E-05	15.885057 47	2.39E-04
hsa05215:Prostate cancer	6	18.75	2.79E-05	15.704545 45	2.39E-04
hsa04014:Ras signaling	8	25	3.08E-05	8.1533923	2.51E-04

pathway				3	
hsa04932:Non-alcoholic fatty liver disease (NAFLD)	7	21.875	3.09E-05	10.67770419	2.40E-04
hsa04660:T cell receptor signaling pathway	6	18.75	5.96E-05	13.41747573	4.42E-04
hsa05146:Amoebiasis	6	18.75	6.85E-05	13.03773585	4.85E-04
hsa04668:TNF signaling pathway	6	18.75	6.85E-05	13.03773585	4.85E-04
hsa04620:Toll-like receptor signaling pathway	6	18.75	6.85E-05	13.03773585	4.85E-04
hsa04931:Insulin resistance	6	18.75	7.49E-05	12.7962963	5.08E-04
hsa04020:Calcium signaling pathway	7	21.875	8.04E-05	9.00744879	5.24E-04
hsa04919:Thyroid hormone signaling pathway	6	18.75	9.69E-05	12.12280702	6.07E-04
hsa04370:VEGF signaling pathway	5	15.625	1.11E-04	18.87978142	6.68E-04
hsa04650:Natural killer cell mediated cytotoxicity	6	18.75	1.34E-04	11.32786885	7.78E-04
hsa05214:Glioma	5	15.625	1.42E-04	17.71794872	7.98E-04
hsa04728:Dopaminergic	6	18.75	1.68E-04	10.796875	9.10E-04

synapse					
hsa04664:Fc epsilon RI signaling pathway	5	15.625	1.69E-04	16.93627451	8.90E-04
hsa04510:Focal adhesion	7	21.875	1.75E-04	7.826860841	8.89E-04
hsa05140:Leishmaniasis	5	15.625	2.00E-04	16.22065728	9.89E-04
hsa04261:Adrenergic signaling in cardiomyocytes	6	18.75	3.10E-04	9.465753425	0.001485797
hsa05143:African trypanosomiasis	4	12.5	3.33E-04	27.91919192	0.001550987
hsa04151:PI3K-Akt signaling pathway	8	25	4.37E-04	5.341062802	0.001978874
hsa04540:Gap junction	5	15.625	4.57E-04	13.08712121	0.00201339
hsa05231:Choline metabolism in cancer	5	15.625	7.71E-04	11.40264026	0.003303933
hsa05168:Herpes simplex infection	6	18.75	8.73E-04	7.551912568	0.00364565
hsa05206:MicroRNAs in cancer	7	21.875	9.94E-04	5.657309942	0.004043799
hsa05014:Amyotrophic lateral sclerosis (ALS)	4	12.5	0.001141097	18.42666667	0.004528862
hsa05213:Endometrial cancer	4	12.5	0.001279418	17.71794872	0.004956215
hsa04270:Vascular smooth muscle contraction	5	15.625	0.001423682	9.677871148	0.005386038

hsa04722:Neurotrophin signaling pathway	5	15.625	0.001468513	9.597222222	0.00542938
hsa04621:NOD-like receptor signaling pathway	4	12.5	0.001506024	16.75151515	0.005444391
hsa04068:FoxO signaling pathway	5	15.625	0.002204282	8.594527363	0.007788956
hsa05321:Inflammatory bowel disease (IBD)	4	12.5	0.0023319	14.39583333	0.008063982
hsa04910:Insulin signaling pathway	5	15.625	0.002454175	8.345410628	0.008309495
hsa05031:Amphetamine addiction	4	12.5	0.002546779	13.95959596	0.008446868
hsa04662:B cell receptor signaling pathway	4	12.5	0.002891492	13.352657	0.009395503
hsa04917:Prolactin signaling pathway	4	12.5	0.003136549	12.97652582	0.009990174
hsa05220:Chronic myeloid leukemia	4	12.5	0.003263731	12.7962963	0.010194947
hsa04921:Oxytocin signaling pathway	5	15.625	0.004002115	7.289029536	0.012257345
hsa04022:cGMP-PKG signaling pathway	5	15.625	0.004773535	6.937751004	0.014339698
hsa05010:Alzheimer's disease	5	15.625	0.004981037	6.85515873	0.014689904
hsa04666:Fc gamma R-mediated phagocytosis	4	12.5	0.005041399	10.96825397	0.014603504

hsa04350:TGF-beta signaling pathway	4	12.5	0.005041399	10.96825397	0.014603504
hsa04970:Salivary secretion	4	12.5	0.005384397	10.71317829	0.015320517
hsa04912:GnRH signaling pathway	4	12.5	0.006302571	10.12454212	0.017611523
hsa04062:Chemokine signaling pathway	5	15.625	0.007128999	6.191756272	0.019571844
hsa05169:Epstein-Barr virus infection	5	15.625	0.007678262	6.061403509	0.020722057
hsa04915:Estrogen signaling pathway	4	12.5	0.007956612	9.306397306	0.021119929
hsa04916:Melanogenesis	4	12.5	0.008179719	9.213333333	0.021361715
hsa04015:Rap1 signaling pathway	5	15.625	0.010847276	5.484126984	0.027824078
hsa04726:Serotonergic synapse	4	12.5	0.010879979	8.3003003	0.027477231
hsa04960:Aldosterone-regulated sodium reabsorption	3	9.375	0.011447842	17.71794872	0.028460402
hsa05219:Bladder cancer	3	9.375	0.012602947	16.85365854	0.030837769
hsa04930:Type II diabetes mellitus	3	9.375	0.017025918	14.39583333	0.040917264
hsa05162:Measles	4	12.5	0.017678413	6.927318296	0.041854125
hsa05144:Malaria	3	9.375	0.017704652	14.10204082	0.04132073

hsa05166:HTLV-I infection	5	15.625	0.021082459	4.498697917	0.048406082
hsa05134:Legionellosis	3	9.375	0.021267523	12.7962963	0.048154093
hsa05221:Acute myeloid leukemia	3	9.375	0.022769775	12.33928571	0.050807993
hsa04730:Long-term depression	3	9.375	0.025902159	11.51666667	0.056914716
hsa05230:Central carbon metabolism in cancer	3	9.375	0.029199956	10.796875	0.063191661
hsa05211:Renal cell carcinoma	3	9.375	0.030049546	10.63076923	0.064158378
hsa04720:Long-term potentiation	3	9.375	0.030909007	10.46969697	0.065120658
hsa05120:Epithelial cell signaling in Helicobacter pylori infection	3	9.375	0.031778248	10.31343284	0.066078518
hsa04610:Complement and coagulation cascades	3	9.375	0.033545706	10.01449275	0.06882189
hsa04622:RIG-I-like receptor signaling pathway	3	9.375	0.034443742	9.871428571	0.069767044
hsa04920:Adipocytokine signaling pathway	3	9.375	0.034443742	9.871428571	0.069767044
hsa05218:Melanoma	3	9.375	0.035351197	9.732394366	0.070707771

hsa04520:Adherens junction	3	9.375	0.035351197	9.732394366	0.070707771
hsa05133:Pertussis	3	9.375	0.039073435	9.213333333	0.077074387
hsa04911:Insulin secretion	3	9.375	0.048995722	8.129411765	0.095036614
hsa05222:Small cell lung cancer	3	9.375	0.048995722	8.129411765	0.095036614
